# A seed-extended algorithm for detecting protein complexes based on density and modularity with topological structure and GO annotations

**DOI:** 10.1186/s12864-019-5956-y

**Published:** 2019-08-07

**Authors:** Rongquan Wang, Caixia Wang, Liyan Sun, Guixia Liu

**Affiliations:** 1 College of Computer Science and Technology, Jilin University, No. 2699 Qianjin Street, Changchun, 130012 China; 2 Key Laboratory of Symbolic Computation and Knowledge Engineering of Ministry of Education, Jilin University, No. 2699 Qianjin Street, Changchun, 130012 China; 3 School of International Economics, China Foreign Affairs University, 24 Zhanlanguan Road, Xicheng District, Beijing, 100037 China

**Keywords:** Graph clustering algorithms, Protein complex, Protein-protein interaction networks, Density, Modularity, functional properties

## Abstract

**Background:**

The detection of protein complexes is of great significance for researching mechanisms underlying complex diseases and developing new drugs. Thus, various computational algorithms have been proposed for protein complex detection. However, most of these methods are based on only topological information and are sensitive to the reliability of interactions. As a result, their performance is affected by false-positive interactions in PPINs. Moreover, these methods consider only density and modularity and ignore protein complexes with various densities and modularities.

**Results:**

To address these challenges, we propose an algorithm to exploit protein complexes in PPINs by a Seed-Extended algorithm based on Density and Modularity with Topological structure and GO annotations, named SE-DMTG to improve the accuracy of protein complex detection. First, we use common neighbors and GO annotations to construct a weighted PPIN. Second, we define a new seed selection strategy to select seed nodes. Third, we design a new fitness function to detect protein complexes with various densities and modularities. We compare the performance of SE-DMTG with that of thirteen state-of-the-art algorithms on several real datasets.

**Conclusion:**

The experimental results show that SE-DMTG not only outperforms some classical algorithms in yeast PPINs in terms of the F-measure and Jaccard but also achieves an ideal performance in terms of functional enrichment. Furthermore, we apply SE-DMTG to PPINs of several other species and demonstrate the outstanding accuracy and matching ratio in detecting protein complexes compared with other algorithms.

## Background

A protein complex is a group of proteins that interact with each other to perform different cellular functions [1]. The detection of protein complexes from protein-protein interaction networks (PPINs) plays an important role in the realization of the cell function in the proteomics era. Specifically, protein complexes contribute to the study of protein interaction network [2], function, diseases [3], etc. Protein complexes help researchers to fully study the causes of various diseases and further develop new drugs. Research on protein complexes is helpful to analyze the different stages of diseases [4]. Current studies have shown that disease genes tend to be highly connected among themselves in disease networks. These highly connected subgraphs could be disease protein complexes and investigation of the cause and effect of these complexes in disease networks could contribute to providing the search space for bioinformaticists, enhance the analysis process [5, 6] and help medical researchers to design new drugs. As a result, the detection of protein complexes plays an indispensable role in complex diseases.

During the past decade, because of the development of high-throughput techniques such as yeast-two-hybrid [7], mass spectrometry [8], and protein chip technologies [9], the number of available PPINs has rapidly increased and have been collected from different public databases. In general, a PPIN can be naturally represented in the form of a network, which not only provides a people the panoramic scope of PPIs on a proteomics scale but also help us to understand the basic organization of cell machinery based on the whole network. How to use PPINs to analyze biological systems remains a meaningful task [10]. Although most of PPINs are missing and inaccurate [11, 12], they reveal biological processes and inherent organizational structures within cells [13–15]. How to accurately discover biological protein complexes is a main subject in biology and bioinformatics. In biology, there are some experimental methods have been designed to detect protein complexes in PPINs, including TAP-ms [16], Co-IP [17–19] and the two-hybrid system [13, 20]. However, biological experimental methods have their own shortcomings; for example, they are time-consuming, relatively expensive and inefficient. Thus, the use of to provide computational algorithms to improve the effectiveness of protein complex detection in PPINs is appealing.

To overcome these experimental constraints, various computational methods have been developed to improve the effectiveness of protein complex detection in PPINs. Some researchers have shown that a protein complex in a PPIN is a molecular structure consisting of both function and structure [21]. Furthermore, some related empirical studies on PPINs also support this point and indicate that modular components in these networks do exist [22]. These results have two implications: one is that these modules are composed closely related proteins and these proteins could have many common neighbor from the perspective of network topology; the other is that proteins in the same modules perform similar functions together in terms of biology. Thus, many researchers believe that proteins in the same complex generally implement the same or similar function and tend to interact with each other [23]. Generally, a PPIN is usually modeled as an undirected graph, where the nodes represent proteins and the edges correspond to protein-protein interactions. Therefore, protein complexes can be detected by mining the modular structures (i.e., dense subgraphs or subnetworks) from PPINs [24]. Based on this idea, the problem of detecting protein complexes in PPINs can be computationally addressed via graph clustering methods, where the resulting biological subgraphs or clusters are considered to be protein complexes. Herein, clustering consists of grouping nodes into groups (also called *clusters or communities*) such that the nodes in the same cluster are more similar to each other than the nodes in the other clusters [25]. Therefore, to overcome the disadvantages of the experimental methods, a series of graph clustering algorithms based on machine learning and data mining are developed as an compensatory choice to detect protein complexes.

### Related work

Up to now, a variety of computational algorithms for detecting protein complexes have been proposed. We first try to make a brief classification of relation work. They mainly include Approaches based on cliques or dense subgraphs, Approaches based on core-attachment structure, Approaches based on hierarchical clustering, Approaches based on model, Approaches based on supervised learning. We will further discuss these methods in the following sections.

#### Approaches based on cliques or dense subgraphs

A large number of existing algorithms suppose protein complexes correspond to k-cliques or highly dense subgraphs. Thus, in the past decade a series of algorithms based on cliques or dense subgraphs have been proposed for detecting protein complexes from PPINs. Until now, many protein complexes detection algorithms also belong to this category. For example, adamcsek et al. [26] provide an application called CFinder to find the k-clique percolation clusters as protein complexes in PPINs. Another example is CMC [27], which first mines the maximal cliques from weighted PPIN, and then removes or merges some highly overlapping maximal cliques. However, this kind of methods require a protein complex to be k-clique or clique. Consequently, some researchers try to discover dense subgraph by using a heuristical searching strategy in a PPIN. For instance, MCODE [28] is one of the earliest this kind methods, which detects protein complexes based on seed-extend method and subgraph with highly density in a PPIN. Several years later, Altaf-UI-Amin et al. [29] propose DPClus, unlike MCODE, DPClus detect densely subgraphs as protein complexes based on the concepts of density and periphery. Following the DPClus, based on the diameter and density, Li et al. [30] present a improved clustering algorithm called IPCA. Several years later, a fast, memory-efficient cluster algorithm SPICi [31] is presented. This cluster algorithm uses density and support function for clustering larger networks.

In fact, approaches based on cliques or dense subgraphs are effective to detect the k-cliques or highly density protein complexes, but they fail to detect either the sparsely subgraph or the relatively peripheral proteins. How to tackle these challenges will be emphasis for further study.

#### Approaches based on core-attachment structure

Most of approaches based on cliques or dense subgraphs mainly focus on the assumption that the highly connected subgraphs may be protein complexes, but these methods ignore the inherent organization of protein complexes. Gavin et al. [14] recently have demonstrated that protein complexes consist a core and some attachments, in which proteins in the core are highly interconnected, and some attachments or protein modules often interact with their core sparsely and assist their core in performing subordinate functions. Employing the core-attachment structure, some outstanding detection algorithms are developed. They have mainly two stages: the first stage is identifying all dense subgraphs and letting them to be the protein complex cores and the second stage is to extend all complex cores by adding peripheral proteins into its core. For example, Wu et al. [32] develop the algorithm named COACH, which first mines some dense subgraphs as protein complex cores and then identifies peripheral proteins. And then peripheral proteins is cooperating with their protein complex core to form a protein complex. Recently, Peng et al. [33] propose another algorithm called WPNCA, which is a new algorithm by using the PageRank-Nibble algorithm and core-attachment structure. Experiments results show that WPNCA is superior to other state-of-the-art algorithm in detecting complexes.

Generally speaking, identified complexes with core-attachment structures have a larger size. In fact, the real protein complexes have a smaller size. It is a directions for further research in the future.

#### Approaches based on model

Up to now, approaches based on model in protein complexes detection are very popular in protein complexes detection. That because they show an excellent performance. Unlike most of algorithms that we mentioned above, approaches based on model focus predominantly on seeking to some relation model or graph pattern to predict protein complexes. It is a new way to discover protein complexes. Markov clustering (MCL) [34] is one of the most popular model by using the random walk strategy in a PPIN, and it has two basic operators called expansion and inflation. MCL can tolerate more noises than other types of algorithms. However, its result depends on the parameter inflation and it does not detect overlapping protein complexes. In fact, overlapping protein complexes takes up a large proportion of protein complexes. Based on this fact, Nepusz et al. [35] introduce a novel method (called ClusterONE) to predict overlapping protein complexes. ClusterONE introduces a cohesiveness (also called graph modularity) to assess the quality of protein complexes for the first time. On the basis of ClusterONE, we introduce CALM [36], a improved method, to detect protein complexes. Firstly, we identify overlapping nodes and seed nodes by calculating node degree and betweenness, then uses a greedy local research approach based on core-attachment and local modularity structure to produce detected protein complexes.

Although the algorithms based on model have good performance for the detection of protein complexes, their accuracy need to be improved by employing network topological features. For example, they could take multiple network topological property or biological informations into account.

#### Approaches based on hierarchical clustering

Recently, due to the form of a tree [37] in PPINs and the nature of modularity [38] in biological networks, some traditional hierarchical clustering algorithms are tried to detect protein complexes in the PPINs. The major difference among them is how to construct the hierarchical structure. More specifically, the key is how to measure the similarity of nodes. Next we introduce some representative algorithms.

Generally, traditional hierarchical clustering algorithms can not be use directly in PPINs with false positives. To overcome this challenge, based on the edge clustering coefficients and *λ*-module, Li et al. [39, 40] propose a new fast hierarchical algorithm for identifying protein complexes, named FAG-EC. Wang et al. modify FAG-EC and propose HC-PIN [41] to identify overlapping and hierarchical functional modules in a PPIN.

In summary, approaches based on hierarchical clustering can provide a global perspective to look at the hierarchical modular organization of a PPIN. What’s more, they are easy to implement and understand. However, most of them can not identify overlapping clusters and are sensitive to the noisiness of the PPINs [42]. Thus, their accuracies are limited. In practice, their performance is deficient in some cases.

#### Approaches based on supervised learning

The aforementioned various computational clustering algorithms are unsupervised-based clustering and they are used for finding protein complexes. All of these unsupervised clustering algorithms only consider one of the multiple topological structure of protein complexes and do not use the known complexes, thus they may ignore complexes with other types of topological structure.

To tackle the defect, with the development of supervised learning algorithms, some researchers utilize the information of known complexes to detect protein complexes from the PPINs. Supervised learning algorithms generally contain three main steps: (1) extract useful features from the known complexes; (2) train a supervised model by distinguishing the real complexes from random subgraphs based on the extracted features; (3) detect protein complexes from the PPINs by using the trained model as fitness evaluating function. So far ClusterEPs [43] is the best among them. It uses emerging patterns to measure the possibility of a subgraph being a complex.

Unfortunately, there is no appropriate feature selection method and the PPINs always have a considerable number of noise. Moreover, the number of known protein complexes is available for training is too small. These disadvantages make the trained model imprecise [44]. Meanwhile, some features are often related to the specific mapping PPINs, so these extracted features may be unique and not universal. As a result, their performance could decrease [45]. Therefore, how to overcome these issues is critical for further improving the accuracy of detection protein complexes.

### Our work

The above algorithms have been shown to detect protein complexes effectively. Furthermore, proteins in the same complex generally possess high functional similarity; thus, protein constituting a complex possibly have similar function. Based on the strengths and weaknesses of the relative works and considering the fact that high-throughput PPINs are noisy and incomplete. Furthermore, proteins in the same protein complex generally possess high functional similarity and more neighbors, proteins constituting a protein complex possibly have similar function and more the same common neighbors. In this paper, we first integrate both common neighbors and GO annotations to construct a weighted PPIN. According to some evidence and research [30, 35, 46], the density-based algorithms and modularity-based algorithms have outstanding performance in PPINs. Thus, we define a new model to quantitatively assess protein complex detection by considering both the density and modularity of a subgraph, and we propose a new graph clustering method based on seed-extend algorithm, namely (SE-DMTG), to detect protein complexes of various dense and modularity. In this process, we grow each seed node to a subgraph until this subgraph is a locally optimal cluster. Furthermore, we remove redundant detected complexes and treat the derived complexes as finally identified protein complexes. Finally, to validate the performance of SE-DMTG, we apply it to PPINs of three different species and compare the results, in terms of the F-measure and Jaccard with those of some representative state-of-the-art algorithms by using several known protein complex datasets that are widely used in biological experiments. The experimental results demonstrate that SE-DMTG outperforms the other competing algorithms in terms of accuracy and matching with known complexes. In addition, these identified protein complexes are subjected to functional enrichment analysis to ascertain their biological significance.

## Results

### Protein-protein interactions datasets selection selection

For performance testing, we carry out all the experiments on three species PPINs: *S*.*cerevisiaecerevisiae* (Yeast), *Homosapiens* (Human) and *Musmusculus* (Mouse). For yeast, we mainly tested three real yeast PPINs. They are Krogan core [15], DIP [55] and combined6, where combined6 [27] is generated by six individual experiments, including interactions characterized by mass spectrometry technique (2002) [56], Gavin et al. (2002, 2006) [14, 57] and Krogan et al. (2006) [15], and interactions produced using two-hybrid techniques [7, 13]. For human, we use two PPINs, which consists of DIP (version Hsapi20170205 on 9/5/2019) [58] and a combined dataset from HPRD (Human Protein Reference Database, 7/2010) [59] and BioGRID (version 3.2.109) [60], namely, HPRD+BioGRID, which is downloaded from Ref [61]. For the mouse, the PPIN of Mus musculus is also obtained from Biogrid (version 3.5.172) [62]: we download Biogrid Mus musculus (BIOGRID-ORGANISM-Mus _musculus-3.5.172.tab.txt), and then we extract the related of mouse file (Biogrid UNIPROT.tab.txt,14/5/2019). Note that, we use all the unweight PPINs to test all algorithms and we remove all self-connecting interactions and repeated interactions. The detail information of these datasets is listed in Table 2.

**Table 1:** Summary of metrics or scores

Symbol	Description
*PPINs*	Protein-protein interaction networks
*G*=(*V,E*)	Graph G with vertex set *V*, edge set *E* and *W* is weight matrix
*N*	Number of vertices in a graph
*M*	Number of edges in a graph
*v*	A vertex in *V*
(*v,u*)	Edge between vertices *v* and *u* in *E*
*N*(*v*)	*N*(*v*) stands for the set of all vertex *v*’neighbors
*CN*(*v,u*)	The weight of edge (*v,u*) according to common neighbors (CN) namely, Eq. (7)
*GO*(*v,u*)	The weight of edge (*v,u*) according to Gene Ontology (GO) namely, Eq.(8)
*w*(*v,u*)	The weight of edge (*v,u*) according to both *CN*(*v,u*) and *GO*(*v,u*), see Eq. (9)
*d*_*w*_(*v*)	The weight degree of vertex *v*
*NGCC*(*v*)	The Neighborhood Graph Clustering Coefficient of vertex *v*
*Score*(*v*)	The priority of vertex *v* is used as seed according to Eq. (12)
*SG*	A subgraph in Graph *G*
*D*(*SG*)	The density of subgraph (SG) according to Eq. (13)
*M*(*SG*)	The modularity of subgraph (SG) according to Eq. (16)
*F*(*SG*)	The fitness of subgraph (SG) according to Eq. (17)
*Neighbor*(*SG*)	The neighbor of the cluster *SG*
*inner*_*nodes*(*SG*)	The inner nodes in the cluster *SG*
*weight*_*avg*_(*SG*)	The average weighted interactions within the cluster *SG* according to Eq. (18)

*Neighbor*(*SG*), the set includes the neighbor node connects to at least one edge with any protein of the cluster *SG* but not belongs to *SG*; *inner*_*nodes*(*SG*), the set includes the inner node belongs to the cluster *SG*, but it connects to at least one node which is the neighbor of *SG*;

**Table 2:** Statistics on the used datasets of PPINs

SP	Name	N	E	D
Yeast	Krogan-Core	2708	7123	0.00194
	DIP	4930	17201	0.00141
	combined6	4671	20461	0.00187
Homo Sapiens	DIP	4615	6892	0.00064
	HPRD+BioGRID	14398	139020	0.00134
Mus musculus	BioGRID	6142	16725	0.00088

SP, the name of species; Name, the name of protein complex data set; N, the number of proteins; E, the number of interactions; D, the density of the PPI network

### Protein complexes selection

To evaluate the performance of different protein complex detection algorithms. For yeast, we employ two known protein complexes sets as standard complexes to evaluate the quality of identified protein complexes by various algorithms in yeast PPINs, namely CYC2008 [63] and SGD [64]. In particular, CYC2008 is constructed from three sources, i.e., 1) MIPS [65], 2) Aloy *et al* [66*], and 3) SGD database [*67*]. For human, we use two standard complexes, which include: 1. CORUM complexes [*68*]. 2. CGPK complexes [*61*] is constructed from four sources, i.e., (1) the Comprehensive Resource of Mammalian protein complexes (CORUM) [*68*]; (2) protein complexes are annotated by GO [*69*]; (3) Proteins Interacting in the Nucleus database (PINdb) [*70*] and (4) KEGG modules [*71*]. For mouse, we use the CORUM complexes [*68*]. Following the work done by Nepusz et al. [*35], we further eliminate those protein complexes that are made up of fewer than three proteins and discard some redundant protein complexes. Finally, the rest of known protein complexes in these databases are used for performance evaluation. The summary of the these standard protein complexes is presented in Table 3.

**Table 3:** Statistics of the gold standard complexes we use

SP	Name	N	P	AS
Yeast	CYC2008	236	1342	6.67
	SGD	238	1170	6.76
Homo Sapiens	CORUM complexes	1824	3167	5.35
	CGPK complexes	2285	6206	8.57
Mus musculus	CORUM complexes	376	1041	4.39

SP, the name of species; Name, the set of protein complexes; N, the number of protein complexes; P, the number of protein coverage; AS, the average size of protein complexes

### Preprocessing

For yeast, we directly use the protein name to represent the proteins in the PPIN and protein complexes. For human and mouse, different PPINs and different standard protein complexes from different sources of datasets are heterogeneous in many aspects. Therefore, we use the *Uniprotid* [72] to represent each protein in this study. As a result, we have a uniform way to represent proteins for both the different PPINs and the standard protein complexes. In the process, we remove all duplication interactions, and proteins is not exist its associated Uniprot accession id.

### Gene Ontology(GO) selection

As for the Gene Ontology (GO) file, for yeast, we use the GO slims which is the cut-down version of GO, it is a subset of the terms in the whole yeast GO. Here, since GO slims of CC include some protein complexes information, we only use GO slims of BP and MF as GO annotations. Moreover, the GO slim information is downloaded from the website (https://www.yeastgenome.org/). Similarly, for human and mouse, we exploit each protein with their associated Biological Process (BP), and Molecular Functions (MF) GO annotation based on the web UniProt [72] (available at https://www.uniprot.org/), and we download these mapping files.

### Evaluation metrics

For the purpose of performance evaluation, This section introduces some evaluation metrics that have been used in this paper. These evaluation metrics calculate the matching degree between identified complexes obtained by different algorithms and standard complexes. Generally, the value of these evaluation metrics falls into the interval between 0.0 and 1.0. The higher the value, the better quality of clustering results and better performance an detecting algorithm has.

1) Precision, Recall, and F-Measure: To evaluate the performance of all algorithms, we match generated complexes with known complexes. First, we introduce the overlap score (*OS*) between the identified protein complexes and known complexes, which is presented as follows [73]:

1 $$ OS(p,g)=\frac{|N_{p}\cap N_{g}|^{2}}{|N_{p}|\cdot |N_{g}|}  $$

Here, |*Np*| is the size of the detected complex, |*Ng*| is the size of the known complex, and |*Np*∩*Ng*| is the common protein number from the detected and known complexes. If *OS*(*p,g*)≥*ω*, we consider *p* and *g* to match each other. In our experiment, we set *ω*=0.2, which is consistent with previous studies [28*,*^29^].

After the overlap score (*OS*) has be defined, we can now give the definition of Precision, Recall, and F-measure as follows [74]:

2 $$ F-measure=\frac{2\times Precision\times Recall}{Precision+ Recall}  $$

where Precision =$\frac {N_{{cp}}}{|P|}$ and Recall =$\frac {N_{{cg}}}{|G|}$. The F-measure is the harmonic mean of Precision and Recall, which can assess the overall performance of the detection algorithms.

2) JaccardI, JaccardS and Jaccard: As we all known, Precision, Recall and F-measure by setting a threshold to judge whether a standard complex and an identified complex are matched or not. It has its limitations because it doesn’t consider the impact of overlapping part on both identified complexes and the corresponding standard complexes [75*]. Therefore, we utilize Jaccard measure for evaluating clustering results [*76*,*^77^*]. It considers the proportion of overlap size in the union set of an identified complex and a standard complex [*75*]. For more details, please refer to Song et al. [*76].

Before we give these metrics, we firstly introduce some notations. Let *I* be the set of identified complexes obtained by a specific identified algorithm, and *S* be the set of standard complexes. Moreover, let *S*_*i*_∈*S* be a standard complex and *I*_*j*_∈*I* represent an identified complex, and then their Jaccard coefficient between them is defined as $Jac(S_{i},I_{j})=\frac {|S_{i}\cap I_{j}|}{|S_{i}\cup I_{j}|}$ [77]. For each identified complex *I*_*j*_, its Jaccard measure is the maximum Jaccard coefficient over all standard complexes i.e, $\phantom {\dot {i}\!}Jac(I_{j}) = max_{S_{i}\in S} Jac(I_{j},S_{i})$. Taking an average over these identified complexes, weighted by complex size, we compute the weighted average Jaccard measure for the all *I* identified complexes.

3 $$ JaccardI=\frac{\sum\nolimits_{I_{j}\in I} |I_{j}|Jac(I_{j})}{\sum\nolimits_{I_{j}\in I}|I_{j}|},  $$

Similarly, for a standard complex *S*_*i*_, its Jaccard measure is $\phantom {\dot {i}\!}Jac(S_{i}) = max_{I_{j}\in I} Jac(S_{i},I_{j})$ and

4 $$ JaccardS=\frac{\sum\nolimits_{S_{i}\in S} |S_{i}|Jac(S_{i})}{\sum\nolimits_{S_{i}\in S}|S_{i}|},  $$

Finally, the Jaccard measure between identified complexes and standard complexes is defined as the harmonic mean of JaccardI and JaccardS.

5 $$ Jaccard=\frac{2\times JaccardI\times JaccardS}{JaccardI + JaccardS}.  $$

According to the definition of Jaccard measure, we can see that Jaccard measure could better evaluate the performance of the identified algorithms than F-measure, especially to compare matching rates of different algorithms.

3) *p*-value: To evaluate the statistical significance of the detected protein complexes, many researchers annotate their main biological functions by using *p*-value [23*,*^78^*]. We calculate the function enrichment test to demonstrate the biological significance of detected protein complexes by different algorithms. In this paper, we use LAGO [*78] to accomplish the function enrichment test with different threshold. Note that, LAGO is a fast tool which finds significant GO terms among a list of gene names, and it computes the significance (*p*-value) via the hypergeometric distribution, and applies (by default) Bonferroni correction. For the details of calculating *p*-value, please refer to [78]. The *p*-value is used for measuring the biological relevance of detected protein complexes and can be denoted as follows.

6 $$ {p}-value=1-\sum\limits_{i=0}^{k-1} \frac{{{F}\choose{i}}{{N-F}\choose{C-i}}}{{{N}\choose{C}}}  $$

where *k* is the number of proteins of the functional group in the protein complex, *N* is the number of proteins in the PPIN. *F* is the size of a functional group in the PPIN, a detected protein complex that contains *C* proteins. Generally, the lower the *p*-value is, the stronger biological significance the protein complex has. The detected protein complex with less than 0.01 is deemed to be meaningful. In additionally, the larger protein complexes possess the smaller *p*-values.

### Comparison with existing algorithms based on known protein complexes

We have experiments on six PPINs to compare our SE-DMTG algorithm with the following state-of-the-art protein complex detection algorithms, including MCODE [28*], MCL [*34*], CFinder [*26*], DPClus [*29*], IPCA [*30*], CMC [*27*], COACH [*32*], HC-PIN [*41*], SPICi [*31*], ClusterONE [*35*], WPNCA [*33*], CALM [*36*], and ClusterEPs [*43]. Here all parameters are set as their authors advised in Table 4. Meanwhile, to evaluate the performance of all algorithms more comprehensively, all the detection algorithms are tested on the three different species that are yeast, human and mouse. Where three yeast PPINs include the Krogan-core, DIP and combined6 dataset. For human, it includes DIP and a combined dataset (HPRD+BioGRID). And we use the BioGRID dataset as mouse PPIN for testing all algorithms. All tested results are presented in Tables 5, 6, 7, 8 and 9. Because the results are similar, we only analyze the results on the yeast in detail and the rest of results are briefly introduced.

**Table 4:** Parameters of each algorithm on datasets

ID	Algorithms	Parameter
1	MCODE	(default setting)
2	MCL	inflation=2(default setting)
3	CFinder	k=3
4	DPClus	*CP*_*in*_=0.5,*d*_*in*_=0.6(default setting)
5	IPCA	S=3,P=2, *T*_*in*_=0.6(default setting)
6	CMC	overlap thres = 0.5 merge thres= 0.5,
		size=3(author suggestions)
7	COACH	w=0.225(default setting)
8	HC-PIN	*λ* = 2.0(default setting)
9	SPICi	density = 0.5, support threshold = 0.5, graph mode = 0(default setting)
10	ClusterONE	s=3,density=auto(default setting)
11	WPNCA	lambda=0.3,size=3(author suggestions)
12	CALM	size=3,weighted= unweighted
		minimum support threshold=0.4,
		maximum support threshold= 0.05,
13	ClusterEPs	Maximum overlap threshold=0.9,
		Maximum size of the clusters = 100
		(author suggestions)
14	SE-DMTG	(default setting and no need parameters)

**Table 5:** Performance comparision on Krogan-core, DIP and combined 6 datasets

Data set	Algorithm	Number	CYC2008			SGD		
			Precision	Recall	F-measure	Precision	Recall	F-measure
Krogan-Core	MCODE	78	**0.7436** ^1 *st*^	0.2839	0.4109	**0.6795** ^1 *st*^	0.2941	0.4105
	MCL	374	0.2727	0.5	0.3529	0.2487	0.4832	0.3283
	CFinder	1396 ^3*rd*^	0.4047	0.5551	0.4681	0.3266	0.5042	0.3965
	DPClus	497	0.2656	0.6144 ^2*nd*^	0.3709	0.2334	0.563 ^2*nd*^	0.33
	IPCA	579	0.5889	0.5339	0.5601 ^3*rd*^	0.4214	0.4916	0.4538
	CMC	**2136** ^1 *st*^	0.0126	0.0636	0.0211	0.0164	0.0798	0.0272
	COACH	348	0.5517	0.5254	0.5383	0.431	0.4832	0.4556 ^3*rd*^
	HC-PIN	167	0.4371	0.3983	0.4168	0.4072	0.4034	0.4053
	SPICi	227	0.3789	0.4322	0.4038	0.3436	0.4034	0.3711
	ClusterONE	243	0.4979	0.4915	0.4947	0.4074	0.4202	0.4137
	WPNCA	374	0.6444 ^2*nd*^	0.5	0.5631 ^2*nd*^	0.4439	0.4412	0.4425
	CALM	1411 ^2*nd*^	0.3671	**0.6314** ^1 *st*^	0.4643	0.3246	**0.584** ^1 *st*^	0.4173
	ClusterEPs	540	0.5333	0.5763	0.554	0.4611 ^3*rd*^	0.542 ^3*rd*^	0.4983 ^2*nd*^
	SE-DMTG	371	0.6253 ^3*rd*^	0.5932 ^3*rd*^	**0.6089** ^1 *st*^	0.5364 ^2*nd*^	0.542 ^3*rd*^	**0.5392** ^1 *st*^
DIP	MCODE	53	0.4151	0.0975	0.1579	0.3585	0.084	0.1362
	MCL	609	0.1741	0.5042	0.2588	0.1511	0.4454	0.2256
	CFinder	2147 ^2*nd*^	0.2399	0.5508	0.3342	0.2068	0.542	0.2994
	DPClus	909	0.1584	0.6653	0.2559	0.1265	0.584	0.208
	IPCA	1242 ^3*rd*^	0.3575	0.6695 ^3*rd*^	0.4661	0.3309	0.6261	0.433
	CMC	1192	0.1695	0.7034 ^2*nd*^	0.2731	0.1518	0.6387 ^3*rd*^	0.2454
	COACH	329	**0.5167** ^1 *st*^	0.5424	0.5292 ^3*rd*^	0.4529 ^2*nd*^	0.5294	0.4882
	HC-PIN	21	0.0476	0.0042	0.0078	0.0476	0.0042	0.0077
	SPICi	402	0.2537	0.4915	0.3347	0.2189	0.4664	0.298
	ClusterONE	341	0.3343	0.428	0.3754	0.305	0.4412	0.3607
	WPNCA	654	0.5015 ^2*nd*^	0.5593	0.5289	0.4465 ^3*rd*^	0.5588	0.4964 ^3*rd*^
	CALM	**2447** ^1 *st*^	0.17	0.6441	0.269	0.1553	0.584	0.2453
	ClusterEPs	728	0.4698 ^3*rd*^	0.6483	0.5448 ^2*nd*^	**0.4657** ^1 *st*^	0.6597 ^2*nd*^	**0.5459** ^1 *st*^
	SE-DMTG	758	0.4644	**0.7585** ^1 *st*^	**0.5761** ^1 *st*^	0.3971	**0.7017** ^1 *st*^	0.5072 ^2*nd*^
combined6	MCODE	63	0.5556 ^2*nd*^	0.1822	0.2744	**0.5238** ^1 *st*^	0.1765	0.264
	MCL	508	0.2126	0.5424	0.3055	0.1969	0.5168	0.2851
	CFinder	**5140** ^1 *st*^	0.1842	0.6949 ^2*nd*^	0.2913	0.1471	**0.6471** ^1 *st*^	0.2397
	DPClus	658	0.2128	0.661	0.3219	0.1839	0.5798	0.2792
	IPCA	2160 ^2*nd*^	0.5296 ^3*rd*^	**0.7034** ^1 *st*^	0.6043 ^2*nd*^	0.4500 ^3*rd*^	0.6176	0.5207 ^2*nd*^
	CMC	892	0.1973	0.6822	0.3061	0.1783	0.6345	0.2783
	COACH	682	0.3959	0.6483	0.4916	0.2918	0.5882	0.3901
	HC-PIN	176	0.4148	0.3602	0.3855	0.3693	0.3361	0.3519
	SPICi	348	0.3506	0.6059	0.4442	0.3132	0.5588	0.4014
	ClusterONE	648	0.2315	0.6229	0.3375	0.2052	0.5882	0.3043
	WPNCA	898	0.4555	0.589	0.5137 ^3*rd*^	0.3697	0.5336	0.4368 ^3*rd*^
	CALM	2064 ^3*rd*^	0.2902	0.6864 ^3*rd*^	0.408	0.2539	0.6218	0.3606
	ClusterEPs	907	0.366	0.6271	0.4623	0.3473	0.6387 ^3*rd*^	0.4499
	SE-DMTG	490	**0.598** ^1 *st*^	0.6864 ^3*rd*^	**0.6392** ^1 *st*^	0.4898 ^2*nd*^	0.6429 ^2*nd*^	**0.556** ^1 *st*^

CYC2008 and SGD are used as standard complexes.

NOTE: The highest value in each row is shown in bold

**Table 6:** Performance comparision on Krogan-core, DIP and combined6 datasets

Data set	Algorithm	Number	CYC2008			SGD		
			JaccardI	JaccardS	Jaccard	JaccardI	JaccardS	Jaccard
Krogan-Core	MCODE	78	0.4492 ^3*rd*^	0.2163	0.292	**0.4001** ^1 *st*^	0.2192	0.2832
	MCL	374	0.2507	0.342	0.2893	0.2195	0.3236	0.2616
	CFinder	1396 ^3*rd*^	0.2913	0.3437	0.3154	0.2311	0.3263	0.2705
	DPClus	497	0.2816	0.4142 ^3*rd*^	0.3352	0.2443	0.3897 ^3*rd*^	0.3003
	IPCA	579	0.4744 ^2*nd*^	0.4016	0.435 ^2*nd*^	0.3403	0.3671	0.3532 ^2*nd*^
	CMC	**2136** ^1 *st*^	0.1065	0.1403	0.1211	0.091	0.1346	0.1086
	COACH	348	0.4206	0.3971	0.4085	0.325	0.3575	0.3405
	HC-PIN	167	0.3543	0.2891	0.3184	0.3152	0.292	0.3032
	SPICi	227	0.3453	0.3383	0.3417	0.2991	0.3165	0.3075
	ClusterONE	243	0.426	0.3568	0.3884 ^3*rd*^	0.3556 ^3*rd*^	0.3244	0.3393 ^3*rd*^
	WPNCA	374	0.3889	0.3646	0.3764	0.2673	0.3239	0.2929
	CALM	1411 ^2*nd*^	0.2728	**0.4495** ^1 *st*^	0.3395	0.2377	**0.4299** ^1 *st*^	0.3061
	ClusterEPs	540	0.3185	0.3034	0.3108	0.2927	0.3034	0.2980
	SE-DMTG	371	**0.5124** ^1 *st*^	0.432 ^2*nd*^	**0.4688** ^1 *st*^	0.3973 ^2*nd*^	0.4044 ^2*nd*^	**0.4008** ^1 *st*^
DIP	MCODE	53	0.188	0.1099	0.1387	0.184	0.1098	0.1375
	MCL	609	0.142	0.33	0.1986	0.1241	0.3031	0.1761
	CFinder	2147 ^2*nd*^	0.1654	0.346	0.2238	0.1544	0.3437	0.2131
	DPClus	909	0.1786	0.3991	0.2468	0.1602	0.3695	0.2235
	IPCA	1242 ^3*rd*^	0.2283	0.4062 ^3*rd*^	0.2923	0.1986	0.3938	0.2641
	CMC	1192	0.2086	0.4344 ^2*nd*^	0.2818	0.1894	0.4081 ^3*rd*^	0.2587
	COACH	329	0.2986 ^3*rd*^	0.3878	0.3374	0.2509	0.3659	0.2977
	HC-PIN	21	0.0097	0.0075	0.0085	0.0142	0.0077	0.01
	SPICi	402	0.2213	0.3303	0.265	0.1944	0.3175	0.2412
	ClusterONE	341	0.2752	0.2909	0.2828	0.2556 ^3*rd*^	0.2918	0.2725
	WPNCA	654	0.2889	0.4059	0.3376 ^3*rd*^	0.2458	0.3922	0.3022 ^3*rd*^
	CALM	**2447** ^1 *st*^	0.1031	0.3773	0.1619	0.0931	0.3519	0.1473
	ClusterEPs	728	0.2992 ^2*nd*^	0.3941	0.3402 ^2*nd*^	**0.2963** ^1 *st*^	0.4107 ^2*nd*^	0.3442 ^2*nd*^
	SE-DMTG	758	**0.3266** ^1 *st*^	**0.4717** ^1 *st*^	**0.386** ^1 *st*^	0.2842 ^2*nd*^	**0.4504** ^1 *st*^	**0.3485** ^1 *st*^
combined6	MCODE	63	0.2309	0.1661	0.1932	0.2089	0.1625	0.1828
	MCL	508	0.2182	0.3799	0.2772	0.1854	0.3456	0.2414
	CFinder	**5140** ^1 *st*^	0.1829	0.4858	0.2658	0.1621	0.4506 ^3*rd*^	0.2384
	DPClus	658	0.2548	0.4742	0.3315	0.2227	0.4261	0.2925
	IPCA	2160 ^2*nd*^	0.3850 ^2*nd*^	0.5025 ^3*rd*^	0.4360 ^2*nd*^	0.3136 ^2*nd*^	0.4556 ^2*nd*^	0.3715 ^2*nd*^
	CMC	892	0.2573	0.5006	0.3399	0.2188	0.4442	0.2932
	COACH	682	0.2872	0.5082 ^2*nd*^	0.3670 ^3*rd*^	0.221	0.4458	0.2955
	HC-PIN	176	0.2606	0.2481	0.2542	0.2225	0.2139	0.2181
	SPICi	348	0.3033 ^3*rd*^	0.4307	0.3560	0.2614 ^3*rd*^	0.382	0.3104 ^3*rd*^
	ClusterONE	648	0.2292	0.4387	0.3011	0.1944	0.3971	0.261
	WPNCA	898	0.2252	0.4339	0.2965	0.181	0.3943	0.2482
	CALM	2064 ^3*rd*^	0.2229	0.476	0.3036	0.1894	0.4255	0.2621
	ClusterEPs	907	0.2542	0.3206	0.2836	0.2533	0.3381	0.2896
	SE-DMTG	490	**0.4679** ^1 *st*^	**0.5208** ^1 *st*^	**0.493** ^1 *st*^	**0.3520** ^1 *st*^	**0.471** ^1 *st*^	**0.4029** ^1 *st*^

CYC2008 and SGD are used as standard complexes.

NOTE: The highest value in each row is shown in bold

**Table 7:** Performance comparision on Homo sapiens (Human) DIP and HPRD+BioGRID datasets

Data set	Algorithm	Number	CORUM complexes			CGPK complexes		
			Precision	Recall	F-measure	Precision	Recall	F-measure
DIP	MCODE	89	0.4157	0.0696	0.1193	0.4382	0.0635	0.1109
	MCL	624	0.1715	0.1606	0.1659	0.1843	0.1549	0.1683
	CFinder	992 ^2*nd*^	0.5030 ^2*nd*^	0.2889 ^3*rd*^	0.367 ^3*rd*^	0.4758	0.2503 ^3*rd*^	0.3281 ^3*rd*^
	DPClus	747	0.249	0.2758	0.2617	0.2544	0.2376	0.2457
	IPCA	904 ^3*rd*^	**0.5796** ^1 *st*^	**0.4063** ^1 *st*^	**0.4777** ^1 *st*^	**0.5719** ^1 *st*^	**0.3295** ^1 *st*^	**0.4181** ^1 *st*^
	CMC	358	0.4832 ^3*rd*^	0.2648	0.3421	0.4860 ^2*nd*^	0.2333	0.3152
	COACH	389	0.4293	0.2303	0.2998	0.4524	0.1996	0.277
	HC-PIN	229	0.1921	0.0521	0.082	0.1878	0.0451	0.0727
	SPICi	369	0.271	0.1804	0.2166	0.2981	0.154	0.2031
	ClusterONE	363	0.3444	0.1623	0.2206	0.3526	0.1453	0.2058
	WPNCA	535	0.4617	0.2072	0.2861	0.4598	0.1834	0.2622
	CALM	**1591** ^1 *st*^	0.2445	0.2363	0.2403	0.2476	0.2232	0.2348
	ClusterEPs	N/A	N/A	N/A	N/A	N/A	N/A	N/A
	SE-DMTG	604	0.4619	0.3317 ^2*nd*^	0.3861 ^2*nd*^	0.4801 ^3*rd*^	0.2998 ^2*nd*^	0.3691 ^2*nd*^
HPRD + BioGRID	MCODE	86	0.1628	0.0148	0.0271	0.1512	0.0162	0.0293
	MCL	1094	0.1088	0.1535	0.1273	0.1353	0.165	0.1487
	CFinder	N/A	N/A	N/A	N/A	N/A	N/A	N/A
	DPClus	1881	0.1691	0.4073	0.2389	0.1898	0.3943	0.2562
	IPCA	**9989** ^1 *st*^	0.2599 ^3*rd*^	0.4594 ^3*rd*^	0.3320 ^3*rd*^	0.2753 ^3*rd*^	0.4639 ^3*rd*^	0.3455 ^3*rd*^
	CMC	N/A	N/A	N/A	N/A	N/A	N/A	N/A
	COACH	4296 ^3*rd*^	0.1925	0.4951 ^2*nd*^	0.2772	0.2146	0.4862 ^2*nd*^	0.2978
	HC-PIN	N/A	N/A	N/A	N/A	N/A	N/A	N/A
	SPICi	1100	0.1409	0.1804	0.1582	0.1618	0.1891	0.1744
	ClusterONE	1763	0.1469	0.2434	0.1832	0.1713	0.2538	0.2046
	WPNCA	2750	0.3222 ^2*nd*^	0.3843	0.3505 ^2*nd*^	0.3578 ^2*nd*^	0.4188	0.3859 ^2*nd*^
	CALM	7810 ^2*nd*^	0.0828	0.2675	0.1265	0.0936	0.2643	0.1382
	ClusterEPs	N/A	N/A	N/A	N/A	N/A	N/A	N/A
	SE-DMTG	2773	**0.4926** ^1 *st*^	**0.6102** ^1 *st*^	**0.5451** ^1 *st*^	**0.5557** ^1 *st*^	**0.6267** ^1 *st*^	**0.5891** ^1 *st*^

CORUM complexes and CGPK complexes are used as standard complexes.

NOTE: The highest value in each row is shown in bold. N/A means that we fails to obtain the results under given program or software

**Table 8:** Performance comparision on DIP and HPRD+BioGRID datasets

Data set	Algorithm	Number	CORUM complexes			CGPK complexes		
			JaccardI	JaccardS	Jaccard	JaccardI	JaccardS	Jaccard
DIP	MCODE	89	0.2585	0.0722	0.1129	0.2671	0.0546	0.0907
	MCL	624	0.1725	0.1736	0.1731	0.1834	0.1356	0.1559
	CFinder	992 ^2*nd*^	0.3448 ^2*nd*^	0.1883	0.2435 ^3*rd*^	0.3269	0.1288	0.1848
	DPClus	747	0.2275	0.1966	0.2109	0.2305	0.1418 ^3*rd*^	0.1756
	IPCA	904 ^3*rd*^	**0.3488** ^1 *st*^	0.2005 ^3*rd*^	0.2546 ^2*nd*^	**0.3455** ^1 *st*^	0.1401	0.1994 ^2*nd*^
	CMC	358	0.3448 ^2*nd*^	0.1881	0.2434	0.3383 ^2*nd*^	0.1347	0.1926 ^3*rd*^
	COACH	389	0.2402	0.171	0.1998	0.2394	0.1234	0.1628
	HC-PIN	229	0.0902	0.0571	0.07	0.1015	0.0478	0.065
	SPICi	369	0.2364	0.1652	0.1945	0.246	0.1208	0.1621
	ClusterONE	363	0.2696	0.135	0.1799	0.2694	0.1029	0.1489
	WPNCA	535	0.2713	0.177	0.2143	0.2697	0.1306	0.176
	CALM	**1591** ^1 *st*^	0.1665	0.2057 ^2*nd*^	0.1841	0.1756	0.1569 ^2*nd*^	0.1657
	ClusterEPs	N/A	N/A	N/A	N/A	N/A	N/A	N/A
	SE-DMTG	604	0.3383 ^3*rd*^	**0.2207** ^1 *st*^	**0.2672** ^1 *st*^	0.3290 ^3*rd*^	**0.1584** ^1 *st*^	**0.2139** ^1 *st*^
HPRD + BioGRID	MCODE	86	0.0969	0.0603	0.0743	0.1027	0.0587	0.0747
	MCL	1094	0.0853	0.1806	0.1158	0.1032	0.1582	0.1249
	CFinder	N/A	N/A	N/A	N/A	N/A	N/A	N/A
	DPClus	1881	0.1943	0.2918	0.2332	0.2123	0.2442	0.2272
	IPCA	**9989** ^1 *st*^	0.2463 ^2*nd*^	0.3139 ^2*nd*^	0.276 ^3*rd*^	0.2548 ^2*nd*^	0.2614	0.2581 ^3*rd*^
	CMC	N/A	N/A	N/A	N/A	N/A	N/A	N/A
	COACH	4296 ^3*rd*^	0.183	0.3114 ^3*rd*^	0.2305	0.1933	0.2641 ^3*rd*^	0.2232
	HC-PIN	N/A	N/A	N/A	N/A	N/A	N/A	N/A
	SPICi	1100	0.171	0.2119	0.1893	0.1944	0.1829	0.1885
	ClusterONE	1763	0.145	0.2166	0.1737	0.1811	0.1932	0.1869
	WPNCA	2750	0.2251 ^3*rd*^	0.3120	0.2615 ^2*nd*^	0.2462 ^3*rd*^	0.2687 ^2*nd*^	0.2570 ^3*rd*^
	CALM	7810 ^2*nd*^	0.0283	0.2456	0.0507	0.0348	0.2001	0.0593
	ClusterEPs	N/A	N/A	N/A	N/A	N/A	N/A	N/A
	SE-DMTG	2773	**0.4409** ^1 *st*^	**0.4046** ^1 *st*^	**0.422** ^1 *st*^	**0.467** ^1 *st*^	**0.3258** ^1 *st*^	**0.3838** ^1 *st*^

CORUM complexes and CGPK complexes are used as standard complexes.

NOTE: The highest value in each row is shown in bold. N/A means that we fails to obtain the results under given program or software

**Table 9:** Performance comparision on Mouse BioGRID datasets

Data set	Algorithm	Number	F-measure			Jaccard		
			Precision	Recall	F-measure	JaccardI	JaccardS	Jaccard
BioGRID	MCODE	45	0.2222 ^2*nd*^	0.0585	0.0926	0.1222	0.0605	0.0809
	MCL	425	0.0635	0.109	0.0803	0.0562	0.1385	0.08
	CFinder	**4420** ^1 *st*^	0.1115	**0.4441** ^1 *st*^	0.1783	0.1566 ^3*rd*^	**0.2874** ^1 *st*^	0.2027 ^2*nd*^
	DPClus	669	0.1166	0.3085	0.1692	0.1389	0.2017	0.1645
	IPCA	1888 ^3*rd*^	0.1372	0.3936 ^3*rd*^	0.2035	0.1526	0.2323 ^3*rd*^	0.1842
	CMC	737	0.1506	0.391	0.2175 ^3*rd*^	0.1703 ^2*nd*^	0.225	0.1939 ^3*rd*^
	COACH	611	0.2029 ^3*rd*^	0.3404	0.2543 ^2*nd*^	0.14	0.2154	0.1697
	HC-PIN	88	0.0682	0.0186	0.0292	0.0276	0.0244	0.0259
	SPICi	288	0.1146	0.1383	0.1253	0.1363	0.1482	0.142
	ClusterONE	327	0.1529	0.1888	0.169	0.1376	0.1349	0.1362
	WPNCA	828	0.1618	0.2553	0.1981	0.0882	0.1831	0.119
	CALM	3596 ^2*nd*^	0.035	0.2899	0.0625	0.0511	0.2135	0.0825
	ClusterEPs	N/A	N/A	N/A	N/A	N/A	N/A	N/A
	SE-DMTG	942	**0.311** ^1 *st*^	0.4309 ^2*nd*^	**0.3613** ^1 *st*^	**0.2133** ^1 *st*^	0.257 ^2*nd*^	**0.2331** ^1 *st*^

CORUM Mouse complexes is used as standard complexes.

NOTE: The highest value in each row is shown in bold

The experimental results of F-measure for different algorithms on yeast PPINs have been summarized in Table 5. As the Table 5 shows, although SE-DMTG doesn’t always obtain best performance on precision or recall, but it always keeps in the top three in all cases. Furthermore, SE-DMTG obtains best F-measure in all three yeast datasets. It means that SE-DMTG makes a better compromise between precision and recall. Therefore, the results of F-measure for SE-DMTG are better than other algorithms. In other words, SE-DMTG is obviously better than other algorithms, especially for the overall accuracy in detected protein complexes. Generally, the performance of SE-DMTG in detecting protein complexes is very promising. The principle reason is that SE-DMTG takes into consideration not only gene ontology data but also the topological structure of the tested PPIN.

We have mentioned the limitations of precision, recall and F-measure earlier in this paper. Furthermore, we employ Jaccard measure to reflect that match ratio between detected protein complex set and standard complex set. Table 6 presents all comparative performance results for different algorithms evaluated based on Jaccard metrics by using CYC2008 and SGD standard complexes, respectively. As can be seen from Table 6, in three yeast PPINs, for Jaccard metric, SE-DMTG consistently outperforms other compared algorithms. That is SE-DMTG has the best value of Jaccard and superior performance. Furthermore, we can see that SE-DMTG clearly dominates the other algorithms in all tested datasets. Therefore, SE-DMTG algorithm can get more competitive value of Jaccard compare to other algorithms, which suggests that SE-DMTG performs better than other classic algorithms in terms of matching ratio on all three datasets. According to the above analysis, we known that the new fitness function we designed is used for dealing with the problem of protein complex detection and seems reasonable to use GO annotations for the detection of protein complexes.

Moreover, we make use of Krogan core dataset to compare the performance of all comparing methods by using CYC2008 and SGD as the standard complexes. As shown in Table 6, the Jaccard of SE-DMTG achieve 0.4688 and 0.4008, respectively, which significantly outperforms other algorithms. Similarly, on DIP dataset, SE-DMTG achieves the highest Jaccard (0.386 and 0.3485). For the combined6 dataset, SE-DMTG also achieves the highest value of Jaccards and the values of Jaccards are 0.5208 and 0.493, respectively. Therefore, it shows that the values of Jaccard in combined6 dataset for SE-DMTG is superior to the results in other datasets. This is mainly because combined6 is more reliable than other two datasets. In other words, PPIN contains multiple source dataset, which maybe lead to more real protein-protein interactions.

To further demonstrate the effectiveness of SE-DMTG algorithm in PPINs on other species, we also carry experiment on the human and mouse PPINs. All comparison results are listed in Tables 7, 8 and 9. Similarly, SE-DMTG also achieves the highest F-measure and Jaccard on other species in most cases. It is noteworthy that the higher F-measure means we can identify protein complexes more accurately and the higher Jaccard represents that detected algorithms have a better matching ratio between detected protein complexes and real protein complexes. In summary, for different species PPINs, SE-DMTG has the best performance over other comparative algorithms in terms of F-measure and Jaccard.

### Biological significance of the detected protein complexes

Due to the incompleteness of the known protein complexes, we should calculate the *p*-value of the detected protein complexes on Cellular component ontologies (CC) by using the tool LAGO (http://go.princeton.edu/cgi-bin/LAGO), which is used for making a functional enrichment analysis [78]. All parameters of LAGO are set default. Because CC includes the information of protein complexes, thus it can better compare the performance of different algorithms. Generally speaking, each protein complex detected by detection algorithm is associated with a *p*-value to show its GO annotations. If the *p*-value of a protein complex is less than 0.01, we consider it biologically significant. In fact, the *p*-values of detected protein complexes have close relationship with their size [33].

Here, to evaluate the functional enrichment of protein complexes detected by different algorithms more comprehensively, we mainly focus on the following three aspects: (1) the number of significant detected protein complexes; (2) the percentage of significant detected protein complexes; (3) the average *p*-value of detected protein complexes. Furthermore, selecting the above approaches to compare with SE-DMTG is because these algorithms are robust performances in most of datasets. More detail you can see their results from Tables 5, 6, 7, 8 and 9. The *p*-values of DPClus, IPCA, CMC, COACH, SPICi, ClusterONE, WPNCA and SE-DMTG are presented in Table 10.

**Table 10:** Function enrichment analysis of the protein complexes identified by SE-DMTG and other algorithms on different datasets

	Algorithms	PC	<E-15	<E-10	<E-5	significant	Avg *p*-value
Yeast							
Krogan core	DPClus	497	37(7.44%)	67(13.48%)	186(37.42%)	231(46.47%)	1.12e-05
	IPCA	579	191(32.99%)	268(46.29%)	435(75.13%)	487(84.11%)	8.24e-06
	CMC	2136	13(0.61%)	109(5.1%)	559(26.17%)	997(46.68%)	2.36e-05
	COACH	348	87(25.0%)	147(42.24%)	253(72.7%)	290(83.33%)	1.04e-05
	SPICi	227	32(14.1%)	54(23.79%)	107(47.14%)	121(53.31%)	4.30e-06
	ClusterONE	243	40(16.46%)	80(32.92%)	153(62.96%)	172(70.78%)	7.54e-06
	WPNCA	374	140(37.43%)	209(55.88%)	311(83.15%)	340(90.9%)	5.00e-06
	SE-DMTG	371	87(23.45%)	162(43.67%)	295(79.52%)	318(85.72%)	4.47e-06
DIP	DPClus	909	49(5.39%)	92(10.12%)	267(29.37%)	353(38.83%)	6.20e-05
	IPCA	1242	345(27.78%)	583(46.94%)	904(72.79%)	1032(83.1%)	1.16e-05
	CMC	1192	63(5.29%)	149(12.5%)	397(33.31%)	553(46.4%)	1.76e-05
	COACH	329	117(35.56%)	184(55.92%)	275(83.58%)	295(89.66%)	5.65e-06
	SPICi	402	37(9.2%)	63(15.67%)	144(35.82%)	189(47.01%)	1.73e-05
	ClusterONE	341	37(10.85%)	72(21.11%)	176(51.61%)	201(58.94%)	3.27e-05
	WPNCA	654	289(44.19%)	420(64.22%)	560(85.63%)	605(92.51%)	6.95e-06
	SE-DMTG	758	171(22.56%)	293(38.65%)	571(75.33%)	633(83.51%)	1.99e-05
combined6	DPClus	658	54(8.21%)	96(14.59%)	225(34.19%)	275(41.79%)	9.87e-06
	IPCA	2160	849(39.31%)	1173(54.31%)	1724(79.82%)	1869(86.53%)	4.15e-06
	CMC	892	71(7.96%)	113(12.67%)	300(33.63%)	400(44.84%)	1.63e-05
	COACH	682	186(27.27%)	273(40.03%)	440(64.52%)	514(75.37%)	9.79e-06
	SPICi	348	37(10.63%)	69(19.83%)	168(48.28%)	203(58.34%)	1.67e-05
	ClusterONE	648	57(8.8%)	105(16.21%)	245(37.81%)	306(47.22%)	1.31e-05
	WPNCA	898	441(49.11%)	593(66.04%)	751(83.63%)	801(89.2%)	3.66e-06
	SE-DMTG	490	154(31.43%)	222(45.31%)	404(82.45%)	423(86.33%)	3.65e-06
Human							
DIP	DPClus	747	11(1.47%)	30(4.01%)	227(30.38%)	336(44.97%)	1.47e-05
	IPCA	904	11(1.22%)	57(6.31%)	359(39.72%)	465(51.45%)	9.76e-06
	CMC	358	16(4.47%)	38(10.62%)	169(47.21%)	231(64.53%)	1.64e-05
	COACH	389	15(3.86%)	45(11.57%)	236(60.67%)	316(81.24%)	1.51e-05
	SPICi	369	12(3.25%)	32(8.67%)	127(34.42%)	191(51.76%)	1.52e-05
	ClusterONE	363	14(3.86%)	36(9.92%)	151(41.6%)	200(55.1%)	1.08e-05
	WPNCA	535	42(7.85%)	114(21.31%)	341(63.74%)	424(79.25%)	1.03e-05
	SE-DMTG	604	38(6.29%)	91(15.06%)	322(53.31%)	413(68.38%)	1.40e-05
HPRD+BioGRID	DPClus	1881	126(6.7%)	240(12.76%)	692(36.79%)	960(51.04%)	1.60e-05
	IPCA	9989	1605(16.07%)	3566(35.7%)	6929(69.37%)	7615(76.24%)	5.06e-06
	CMC	N/A	N/A	N/A	N/A	N/A	N/A
	COACH	4296	1106(25.74%)	1855(43.17%)	3218(74.9%)	3596(83.7%)	7.50e-06
	SPICi	1100	84(7.64%)	152(13.82%)	374(34.0%)	522(47.45%)	1.65e-05
	ClusterONE	1763	123(6.98%)	227(12.88%)	531(30.12%)	695(39.42%)	1.21e-05
	WPNCA	2750	719(26.15%)	1126(40.95%)	1867(67.9%)	2164(78.7%)	1.00e-05
	SE-DMTG	2773	626(22.57%)	1059(38.18%)	1935(69.77%)	2235(80.59%)	1.15e-05
Mouse							
BioGRID	DPClus	669	7(1.05%)	29(4.34%)	182(27.21%)	304(45.45%)	2.67e-05
	IPCA	1888	121(6.41%)	427(22.62%)	767(40.63%)	1069(56.63%)	1.47e-05
	CMC	737	4(0.54%)	30(4.07%)	217(29.44%)	367(49.79%)	2.19e-05
	COACH	611	59(9.66%)	112(18.33%)	313(51.23%)	430(70.38%)	1.64e-05
	SPICi	288	1(0.35%)	18(6.25%)	101(35.07%)	145(50.35%)	1.91e-05
	ClusterONE	327	3(0.92%)	27(8.26%)	121(37.01%)	177(54.14%)	2.42e-05
	WPNCA	828	170(20.53%)	275(33.21%)	525(63.4%)	657(79.34%)	1.23e-05
	SE-DMTG	832	60(7.21%)	140(16.83%)	401(48.2%)	519(62.38%)	2.13e-05

NOTE: The table lists the percentages of protein complexes detected by DPClus, IPCA, COACH, WPNCA and SE-DMTG in PPI network of different species whose *p*-value falls within different value ranges. N/A means that we fails to obtain the results under given program or software

In Table 10, we summarize the results of DPClus, IPCA, CMC, COACH, SPICi, ClusterONE, WPNCA and SE-DMTG by using function enrichment tests with different thresholds of *p*-value. As shown in Table 10, in most cases, SE-DMTG can detect many candidates of protein complexes than other methods such as DPClus, CMC, SPICi and ClusterONE in all PPINs. Furthermore, by analyzing functional enrichment, especially for the number, percentage and average *p*-value of detected protein complexes detected by SE-DMTG have statistical significance to compare with these algorithms mentioned above. As the Table 10 shows, although the number of significant protein complexes detected by IPCA is the most, the percentage and the average *p*-value of significant detected protein complexes is slight lower than SE-DMTG, COACH and WPNCA. Furthermore, the percentage and the average *p*-value of significant protein complexes detected by SE-DMTG from the six PPINs is a bit lower than COACH and WPNCA. It is the third highest among all methods. The major reason is that the size of protein complexes detected by SE-DMTG is smaller than the size of detected protein complexes by COACH and WPNCA. In fact, the smaller detected protein complexes have the larger *p*-values. More detail about the relationship between the size of detected protein complexes and the *p*-value of detected protein complexes. We will discuss in the relationship of the size of identified protein complexes and the *p*-value of significant detected protein complexes section.

### Examples of detected complexes

In Tables 11 and 12, we further reveal the computation results, 18 detected protein complexes with very low *p*-values (≤E-20) detected by our SE-DMTG algorithm in six datasets are presented. You can see that the *p*-value of these detected protein complexes are very low. It demonstrates that the detected protein complexes by SE-DMTG have high statistic significance.

**Table 11:** Eighteen detected protein complexes which have low *p*-value by SE-DMTG on different datasets

ID	Size	Gene Ontology term	*p*-value	Number annotated
			Yeast Krogan core	
2	20	proteasome accessory complex	1.63952e-47	19
16	15	proteasome core complex	1.22974e-36	14
24	14	RSC-type complex	9.83789e-36	14
			Yeast DIP	
8	21	endopeptidase complex	2.47705e-40	19
31	14	core mediator complex	6.85485e-33	13
35	13	mRNA cleavage and polyadenylation specificity factor complex	5.64982e-32	12
			Yeast combined6	
8	29	spliceosomal snRNP complex	4.80656e-51	27
18	22	mediator complex	2.10967e-54	22
65	12	RNA polymerase I complex	3.94103e-33	12
			Human DIP	
6	11	mediator complex	9.44868e-23	10
7	10	eukaryotic 48S preinitiation complex	3.32604e-24	9
47	7	transcription factor TFIIH core complex	7.88064e-22	7
			Human HPRD+BioGRID	
3	61	cytosolic ribosome	4.51244e-134	59
75	38	proteasome complex	3.30322e-95	37
109	31	mitochondrial large ribosomal subunit	3.84718e-73	29
			Mouse BioGRID	
1	29	postsynaptic density	9.9542e-26	22
67	10	PRC1 complex	2.33107e-21	8
118	7	ESC/E(Z) complex	7.92617e-20	7

NOTE: Table 6 presents 18 detected protein complexes which have low *p*-value. The first column and the fourth column show their ID and their *p*-value. The second column presents the size of detected protein complexes. Gene ontology term (in the third column) show the detected complex contains the proteins of the detected complexes, in which the protein with emph style matches the gene ontology. Number annotated (in the fifth column) represents the number of genes from the detected protein complexes that are found within the annotation and within the aspect

**Table 12:** Eighteen detected protein complexes detected by SE-DMTG

ID	Predicted complexes
	Yeast Krogan core
2	**YDL007W, YDL097C, YDL147W, YDR363W-A, YDR394W, YDR427W,**
	**YER021W, YFR004W, YFR052W, YGL048C, YHR027C, YHR200W, YIL075C,**
	**YKL145W, YLR421C, YOR117W, YOR259C, YOR261C, YPR108W**,YFR010W
16	**YBL041W, YER012W, YER094C, YFR050C, YGL011C, YGR135W, YGR253C,**
	**YJL001W, YML092C, YMR314W, YOL038W, YOR157C, YOR362C, YPR103W,** YBR173C
24	**YCR020W-B, YCR052W, YDR303C, YFR037C, YGR275W, YIL126W, YKR008W,**
	**YLR033W, YLR321C, YLR357W, YML127W, YMR033W, YMR091C, YPR034W**
	Yeast DIP
8	**YDL007W, YDL097C, YDL147W, YDR394W, YDR427W, YEL037C, YHR200W,**
	**YKL145W, YER012W, YER021W, YFR004W, YFR052W, YGL004C, YGL048C,**
	**YLR421C, YOR117W, YOR259C, YOR261C, YPR108W,** YBR272C, YFR010W
31	**YBL093C, YBR193C, YBR253W, YDL005C, YER022W, YGR104C, YHR041C,**
	**YHR058C, YLR071C, YMR112C, YNL236W, YOL051W, YOL135C,** YOR140W
35	**YAL043C, YDR195W, YDR301W, YGR156W, YJR093C, YKL059C,**
	**YKR002W, YLR115W, YLR277C, YNL222W, YNL317W, YPR107C,** YMR061W
	Yeast combined6
8	**YBL026W, YBR055C, YBR152W, YDL087C, YDR378C, YDR473C,**
	**YER029C, YER112W, YER172C, YFL017W-A, YGR074W, YGR091W,**
	**YHR165C, YJL203W, YKL173W, YLR147C, YLR275W, YML049C,**
	**YMR240C, YMR288W, YNL147W, YNL286W, YOR159C, YOR308C,**
	**YPL213W, YPR178W, YPR182W,** YDL030W, YOR148C
18	**YBL093C, YBR193C, YBR253W, YCR081W, YDL005C, YDR308C,**
	**YDR443C, YER022W, YGL025C, YGL127C, YGL151W, YGR104C,**
	**YHR041C, YHR058C, YLR071C, YNL236W, YNR010W, YOL051W,**
	**YOL135C, YOR174W, YPR070W, YPR168W**
65	**YBR154C, YDR156W, YJL148W, YJR063W, YNL113W, YNL248C,**
	**YOR210W, YOR340C, YOR341W, YPR010C, YPR110C, YPR187W**
	Human DIP
6	**CCNC, CDK8, MED1, MED10, MED12, MED14, MED16, MED17,**
	**MED24, MED26,** GATA1
7	**EIF3A, EIF3C, EIF3D, EIF3E, EIF3H, EIF3J, EIF3K, EIF3L, EIF3M,** EIF3F
47	**ERCC2, ERCC3, GTF2H1, GTF2H2, GTF2H3, GTF2H4, GTF2H5**
	Human HPRD+BioGRID
3	**RPL10A, RPL10L, RPL11, RPL12, RPL13, RPL14, RPL15, RPL17, RPL18,**
	**RPL18A, RPL19, RPL21, RPL22, RPL23, RPL23A, RPL24, RPL3,**
	**RPL30, RPL31, RPL32, RPL37A, RPL4, RPL5, RPL6, RPL7, RPL7A,**
	**RPL8, RPL9, RPLP0, RPS10, RPS11, RPS12, RPS13, RPS14, RPS15A,**
	**RPS16, RPS18, RPS19, RPS2, RPS20, RPS21, RPS23, RPS24, RPS25, RPS26,**
	**RPS27, RPS27A, RPS27L, RPS28, RPS29, RPS3, RPS3A, RPS4X, RPS5,**
	**RPS6, RPS7, RPS8, RPS9, RPSA,** TSR1, PYM1
75	**PSMA1, PSMA2, PSMA3, PSMA4, PSMA5, PSMA6, PSMA7, PSMA8,**
	**PSMB1, PSMB2, PSMB3, PSMB4, PSMB5, PSMB6, PSMB7, PSMB8, PSMC1,**
	**PSMC2, PSMC3, PSMC4, PSMC5, PSMC6, PSMD1, PSMD11, PSMD12, PSMD13,**
	**PSMD14, PSMD2, PSMD3, PSMD4, PSMD6, PSMD7, PSMD8, PSME1, PSME2,**
	**RAD23B, UBQLN1,** SEM1
109	**MRPL10, MRPL11, MRPL12, MRPL13, MRPL14, MRPL15, MRPL16, MRPL19,**
	**MRPL2, MRPL23, MRPL24, MRPL3, MRPL32, MRPL37, MRPL38, MRPL39,**
	**MRPL4, MRPL40, MRPL41, MRPL42, MRPL44, MRPL45, MRPL50, MRPL51, MRPL52,**
	**MRPL55, MRPL58, MRPL9, MRPS18A, A4, MRPS9**
	Mouse BioGRID
1	**Baiap2, Camk2a, Camk2b, Cnksr2, Dlg1, Dlg2, Dlg4, Dlgap1, Dnm1, Fxr1, Grin1,**
	**Grin2a, Grin2b, Homer1, Iqsec1, Kalrn, Prkcg, Shank1, Shank2,**
	**Shank3, Sptbn1, Syngap1,** Mdk, Cyfip2, Nckap1, Pde4dip, Tnik, Cyfip1, Agap2
67	**Bmi1, Cbx2, Cbx7, Pcgf2, Phc1, Phc2, Ring1, Rnf2,** Aurkb, Rybp
118	**Epop Ezh2 Jarid2, Mtf2 Rbbp4 Suz12,Ezh1**

NOTE: The first column show their ID. The second column presents detected protein complexes by SE-DMTG. In this table proteins in bold are found within the annotation and with the aspect, and the rest is not found

**Fig. 1:**
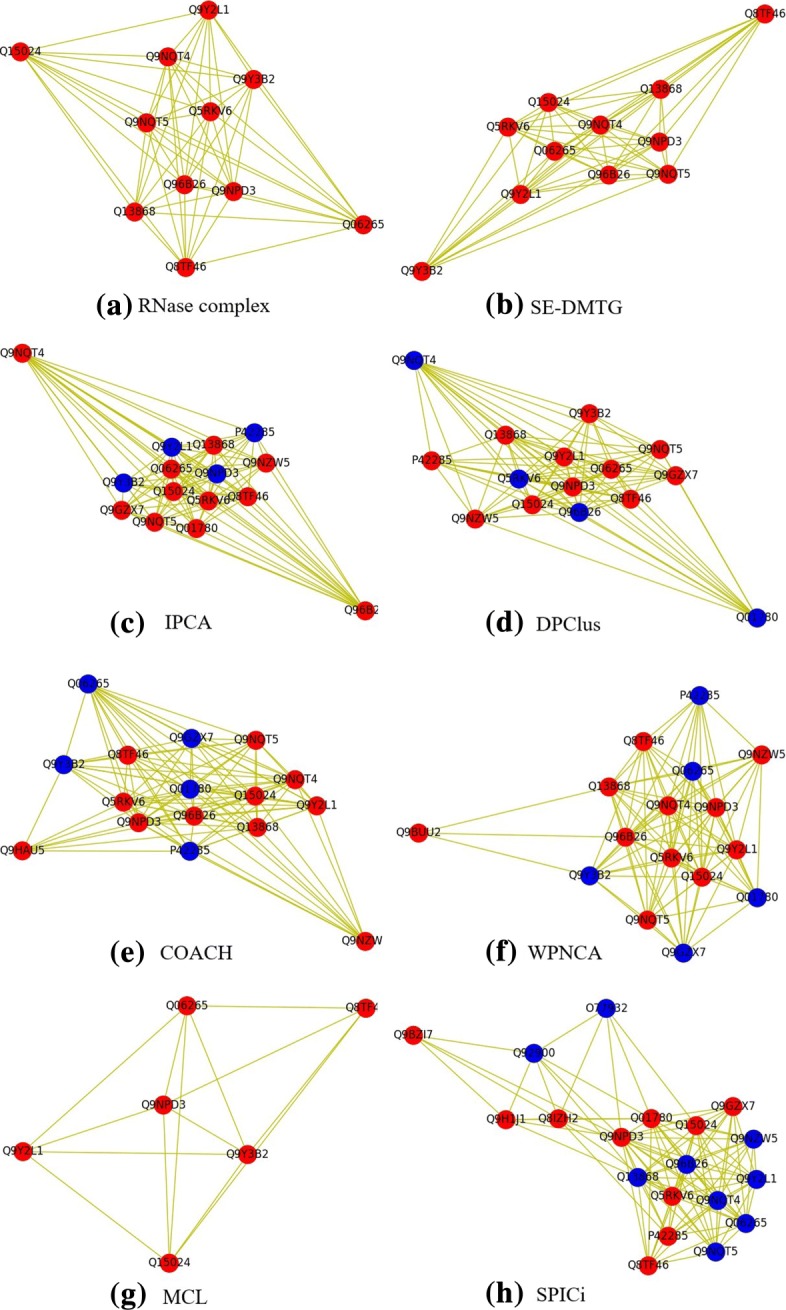
A standard protein complex called ’RNase complex’ which is come from CGPK complexes is detected by different algorithms in HPRD+BioGRID dataset. Fig.**a** shows the real ’RNase complex’ in the PPIN of human HPRD+BioGRID dataset. Fig.**b**-**h** are the protein complexes detected by SE-DMTG, IPCA, DPClus, COACH, WPNCA, MCL and SPICi, respectively. The red nodes represent the accurately detected proteins and the blue nodes represent the proteins that are not inaccurately identified proteins

To further reveal the comparison results obtained by SE-DMTG, we provide with a more vivid description by taking the 391th known protein complex of CGPK complexes-’RNase complex’ as example. As shown in Fig. 1a, the known protein complex has 11 proteins. Meanwhile the detected protein complex obtained by SE-DMTG algorithm also consists of 11 proteins and it successfully match all proteins and its *OS* is 100% which is the highest among all algorithms. This result is shown in Fig. 1b. However, the IPCA, DPClus, COACH, WPNCA, MCL and SPICi just cover 11, 11, 11, 11, 6 and 10 proteins of the real RNase complex, respectively. And for the rest of compared algorithms, their *OS* (see Eq. (1)) is lower than 0.47 or they are not able to get the detected results. So we don’t show them in Fig. 1. However, for the IPCA, DPClus, COACH, WPNCA, MCL and SPICi algorithms, their *OS* value is only 73%,73%,68%,68%,54% and 47%, respectively. This result means that SE-DMTG can detect protein complexes accurately, indicating that the new definition of protein complex is also a good model to characterize the topological structure of the protein complexes. Additionally, from this example we explain that why SE-DMTG could achieve highest F-measure and Jaccard but its the percentage of significant detected protein complexes and the average of *p*-value are slightly lower than COACH and WPNCA. In summary, protein complexes detected by SE-DMTG are more biological significance.

In a word, based on the results of *p*-value test, we have the conclusion that SE-DMTG can detect quite accurately and have good functional enrichments than other thirteen comparative algorithms.

## Discussion

### The relationship between the size of detected protein complexes and the *p*-value of detected protein complexes

**Fig. 2:**
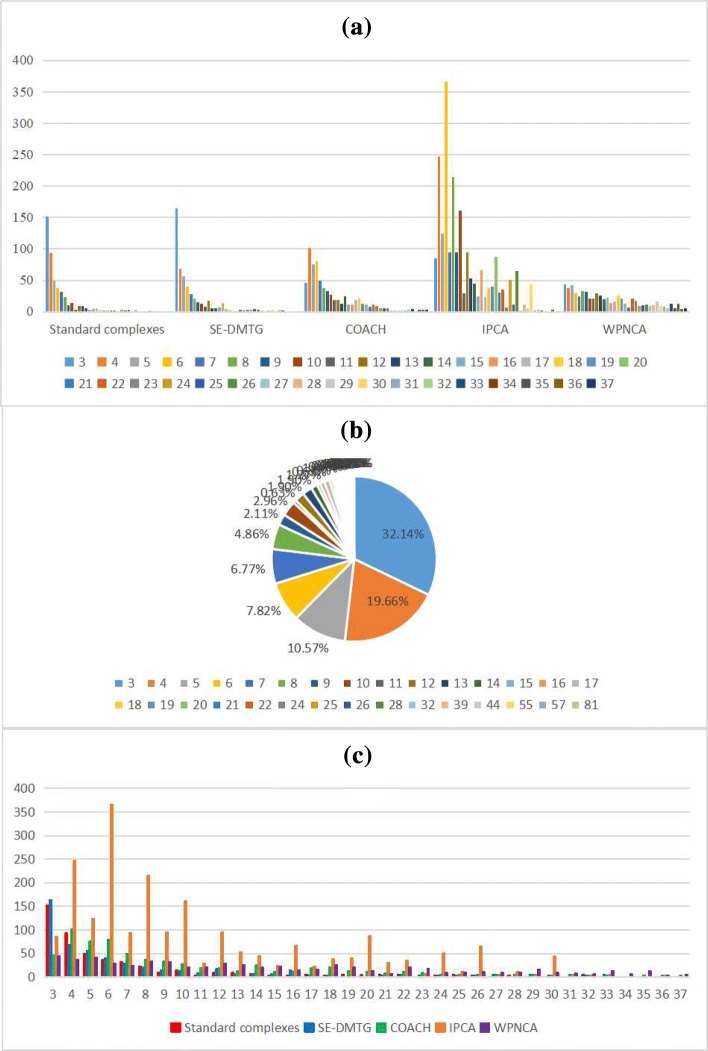
The distribution of the size of protein complexes in the PPIN. In Fig.**a** and **c**, the horizontal axis is the different algorithms and the size of protein complex, and the vertical axis is the number of protein complexes which fall in each size. In Fig. **b**, it is the distribution of the different size of combined standard protein complexes consisting of CYC2008 and SGD complexes

To illustrate the relationship between the size of detected protein complexes and the *p*-value of detected protein complexes, we do some statistical analysis. Because standard complexes and detected protein complexes are resemble ’power law’ distribution. Thus we only display part of the distribution informations in Fig. 2. According to Fig. 2a, the size of most of standard complexes is very smaller. As shown in Fig. 2b, standard complexes whose size is less than or equal to 7 is just 76.96%. Meanwhile, our statistic results show that the average size of the combined standard complexes is 6.38 and the average size of detected protein complexes by SE-DMTG is 6.86. But the average size of detected protein complexes by IPCA, COACH and WPNCA is 10.96, 10.20 and 27.12, respectively. The average size of detected protein complexes by SE-DMTG is similar with standard complexes. However, in Fig. 2c, we found IPCA, COACH and WPNCA detect a larger number of large protein complexes. Additionally, the size of detected protein complexes by SE-DMTG is similar distribution with standard complexes in Fig. 2a and c.

**Fig. 3:**
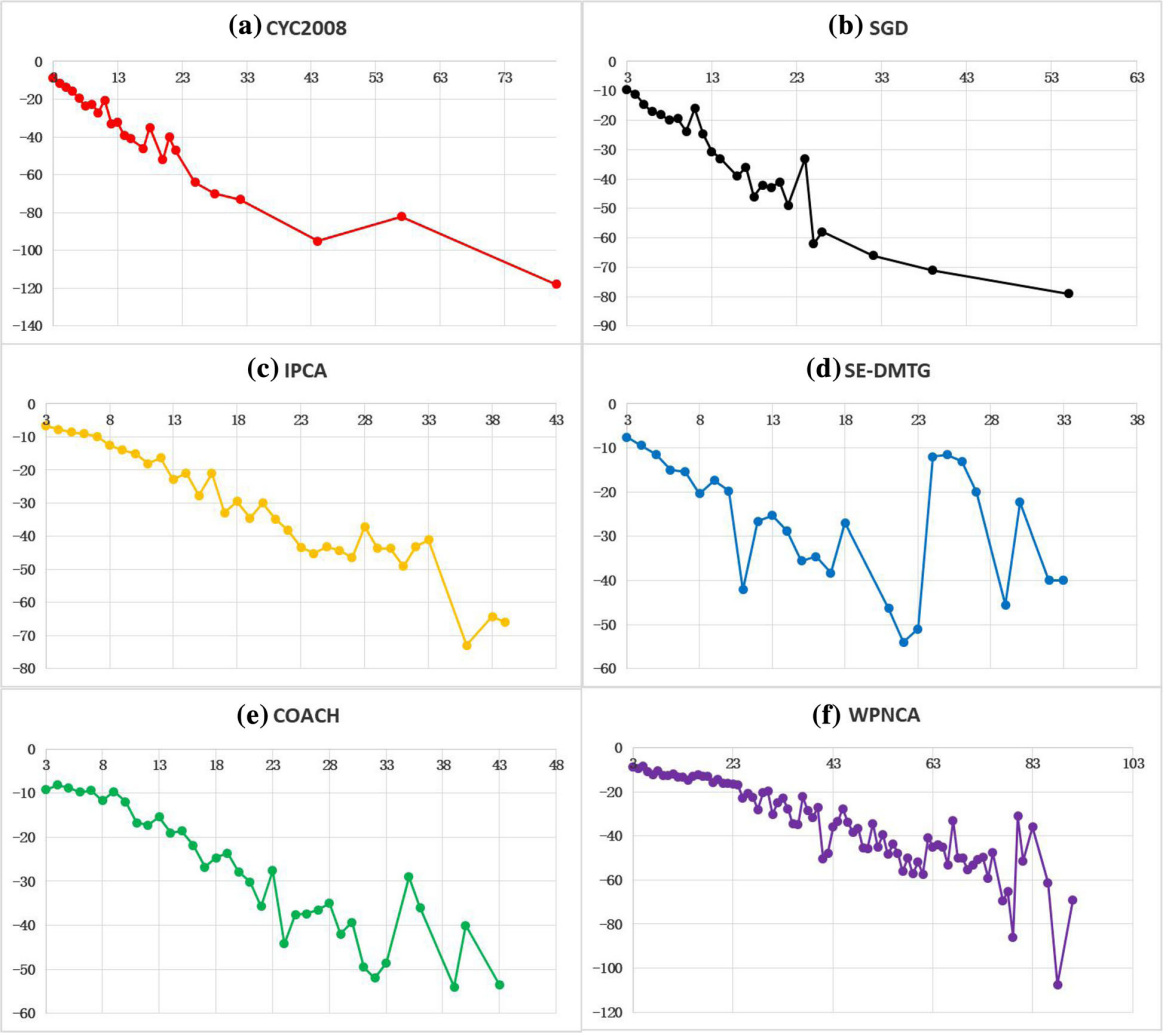
Values of *p*-value (E) for different sizes of standard and detected protein complexes in combined6 dataset. The horizontal axis is the size of protein complexes and the vertical axis is the average *p*-value (E) of this size protein complex. **a** CYC2008 standard protein complexes; **b** SGD standard protein complexes; **c** detected protein complexes by IPCA; **d** detected protein complexes by SE-DMTG; **e** detected protein complexes by COACH; **f** detected protein complexes by WPNCA

Next, we make Fig. 3 to illustrate the relationship of the size of protein complexes with the percentage of significant detected protein complexes and the average *p*-value of detected protein complexes. From Fig. 3, it is obvious that the value of *p*-value (E) decreases gradually with the detected protein complexes whose size increasing. For example, the *p*-value of standard complexes decreases gradually with the size of protein complexes increasing in Fig. 3a. Similarly, for detected protein complexes by IPCA in Fig. 3c, the value of *p*-value decreases gradually when the size of detected protein complexes increases. Therefore, it illustrates that large detected protein complexes have small *p*-value. But in Fig. 2a and b, we know that most of standard complexes and protein complexes by SE-DMTG have small size. Above analysis explains why SE-DMTG has a higher accuracy and matching better with standard complexes according to Tables 5, 6, 7, 8 and 9. However, as for the percentage of significant detected protein complexes and the average *p*-value of detected protein complexes, SE-DMTG is slightly lower than COACH and WPNCA, and it is the third highest among all methods according to Table 10.

All in all, although *p*-value has limitation in evaluating functional significant of detected protein complexes, it also reflects the function enrichment of detected protein complexes in a certain level. Overall, considering the superior accuracy and matching ratio and their strong performance in the function enrichment test, we believe the protein complexes detected by SE-DMTG are more likely to be real protein complexes.

### Computational complexity of SE-DMTG

#### Experimental setup

We implement SE-DMTG in python and execute all the experiments on a 64-bit Window system, whose memory of PC is 12GB and Intel CPU is i7 3.60 GHz. In the meantime all state-of-the-art methods are also executed on the same machine, except SPICi. While SPICi method is used through its web site.

#### Time complexity analysis

In this part, we try to analyze the time complexity of the SE-DMTG algorithm. It is difficult to give the accurately computational complexity of SE-DMGT because it depends on not only the number of detected protein complexes but also their size. Moreover, for each seed, we need to execute an iterative procedure until the current cluster doesn’t changes, Obviously the number of iterations have significant influence for the computational complexity of SE-DMTG. Thus, we only roughly analyze the time complexity. Let *n* and *m* denote the number of nodes and edges in graph *G*, respectively, and let $\overline {k}$ be the average number of neighbors of all the nodes. Then we have $\overline {k}=\frac {\sum \nolimits _{v\in V}N(v)}{n}$, where *N*(*v*) is the number of all neighbors of *v*. In construct a weighted PPIN step, time complexity of calculating the weight of all edge is $O\left (n*\overline {k}\right)=O\left (n*\frac {\sum \nolimits _{v\in V}N(v)}{n}\right)=O\left (\sum \nolimits _{v\in V}N(v)\right)=O(2*m)$. In constructing a seed queue *SQ* and selecting the initial cluster step, according to Eq. (12), the time complexity of we calculating the score of each protein is $O(n*(\overline {k})+1)^{2}=O(n*\left (\frac {\sum \nolimits _{v\in V}N(v)}{n}+1\right)^{2}=\frac {4*m^{2}}{n}+4*m+n$ and the time complexity of sorting all proteins by their *Score*(*v*) is *O*(*n*∗*log*(*n*)). In the generate detected protein complex step, the worst case is that we need calculate the fitness of each protein and its worst time complexity also is $\frac {4*m^{2}}{n}+4*m+n$.

In generating detected protein complexes step, we firstly analysis the time complexity when SE-DMTG iteratively adds proteins to the cluster *SG* from its neighbors. It has three basic phases: (1) obtain all candidate nodes which will be added to the cluster *SG*, whose time complexity is $O(n_{{SG}}*\overline {k})=O\left (n_{{SG}}*\frac {\sum \nolimits _{v\in V}N(v)}{n}\right)=O\left (\frac {2*n_{{SG}}*m}{n}\right)$, where *n*_*SG*_ is the number of the cluster *SG*. (2) find the highest priority vertex according to Eq. (18) then add it into the cluster *SG*. The worst time case is that each candidate node is checked, so the time complexity of this case is $O\left ((N_{{SG}}+N_{{SG}}-1+...+1)*\overline {k}\right)=O\left (\frac {m*N_{{SG}}*(N_{{SG}}-1)}{n}\right)$, where *N*_*SG*_ is the number of neighbors of *SG*. (3) calculate the fitness of graph *SG*, whose time complexity is $O(n_{{SG}}^{2})$. Thus, the time complexity of the whole time when program iteratively add candidate nodes to the cluster *SG* is $O\left (\frac {2*n_{{SG}}*m}{n}+\frac {m*N_{{SG}}*(N_{{SG}}-1)}{n}+n_{{SG}}^{2}\right)$. Meanwhile, we further analyze the time complexity of iteratively removing some inner nodes from *SG*. Similar, it also has three basic calculations: (1) determine the inner nodes which are removed them from the cluster *SG*. Its time complexity is also $O\left (\frac {2*n_{{SG}}*m}{n}\right)$. (2) find a high priority vertex according to Eq. (18) in order to remove it from the cluster *SG*. Its time complexity is also $O\left ((N_{{SG}}+N_{{SG}}-1+...+1)*\overline {k}\right)=O\left (\frac {m*N_{{SG}}*(N_{{SG}}-1)}{n}\right)$. (3) calculate the fitness of graph *SG*. Its time complexity is $O(n_{{SG}}^{2})$. Hence the time complexity of this step is $O\left (\frac {2*n_{{SG}}*m}{n}+\frac {m*N_{{SG}}*(N_{{SG}}-1)}{n}+n_{{SG}}^{2}\right)$.

Suppose *t* is the number of iteractions when we generate a detected protein complex and *N* is the number of detected protein complexes. Finally, the time complexity of Algorithm 2 is $O(N*t*\frac {m}{n}*\left (N_{{SG}}*(N_{{SG}}-1)+3*n_{{SG}}*(1+n_{{SG}})\right)$. Finally, we need to discard some redundant protein complexes whose time complexity is *O*(*PCs*^2^), where *PCs* is the size of candidate identified protein complexes. All in all, the time complexity of the algorithm SE-DMTG is $O(2*m+\frac {4*m^{2}}{n}+4*m+n+n*log(n)+N*t*\frac {m}{n}*\left (N_{{SG}}*(N_{{SG}}-1)+3*n_{{SG}}*(1+n_{{SG}})+len(PCs)^{2}\right)$, where *N,t* and *PCs* are constant. In addition, we assume *N*_*SG*_ and *n*_*SG*_ as variables. To facilitate the intuitive understanding of these variables, we provide Table 13 so that you can get more details.

**Table 13:** Some variables used in SE-DMTG algorithm

Species	Datasets	Number	Average size	Average iterations	Time
Yeast	Krogan core	371	5.77	2.41	3.24 s
	DIP	758	5.48	2.47	14.88 s
	combined6	490	6.86	2.44	11.50 s
Human	DIP	604	4.37	2.40	2.80 s
	HPRD+BioGRID	2773	7.66	2.55	679.01 s
Mouse	BioGRID	942	5.74	2.62	43.41 s

## Conclusion

Many high-throughput experimental techniques and computational algorithms have been developed to identify protein complexes from the PPINs. However, most of these methods are based on the original network or use the topological property alone and are thus limited in terms of not only the quality of protein complex identification but also ignoring other useful biological information, such as functional properties. In our opinion, both topological and functional properties are meaningful and important for identifying protein complexes. We therefore combine common neighbor and functional properties to calculate edge weights and construct weighted PPINs. Moreover, we also propose a new local search heuristic graph clustering algorithm, SE-DMTG, to extract detected protein complexes with various densities and modularities based on a new model. Although models that consider density or modularity have been applied to study PPINs, our model is novel in considering both density and modularity simultaneously.

We evaluate the performance of the proposed SE-DMTG on three PPINs of species under some standard complex datasets and compare the results with those of thirteen competing algorithms. The experimental results show that SE-DMTG is competitive in identifying protein complexes and that adding the topological information and GO information increases the detection accuracy. Meanwhile, the experimental results reveal that SE-DMTG outperforms the current state-of-the-art algorithms in terms of some measures in overall. Furthermore, we analysis the biological significance of detected protein complexes by different methods. The results show that these detected protein complexes by SE-DMTG have biological significant. With the wide application of supervised learning, we will try to design a new algorithm that combines classification model and unsupervised clustering algorithms to improve the performance in the future. Additionally, SE-DMTG is also robust to false positives in experimental data because of the integration of functional properties. Furthermore, SE-DMTG may be extended naturally to other types of biological data fusion to study more comprehensive characteristics of the biological networks and to analyze other forms of complex networks, such as Internet networks, citation networks, ecological networks and social networks.

## Methods

### Preliminaries

Since the interactions among proteins in the PPINs are symmetric, these PPINs could be formulated as a undirected weighted graph *G*=(*V,E*,*W*), where *V* is a set of nodes representing the proteins of the PPINs, *E* is a set of undirected edges corresponding to those interactions, and *W* represents the likelihoods between nodes. In this paper, we obtain the weights by using the topological information and the biological information. The symbols, abbreviations and their interpretation are shown in Table 1.

### Algorithm framework

**Fig. 4:**
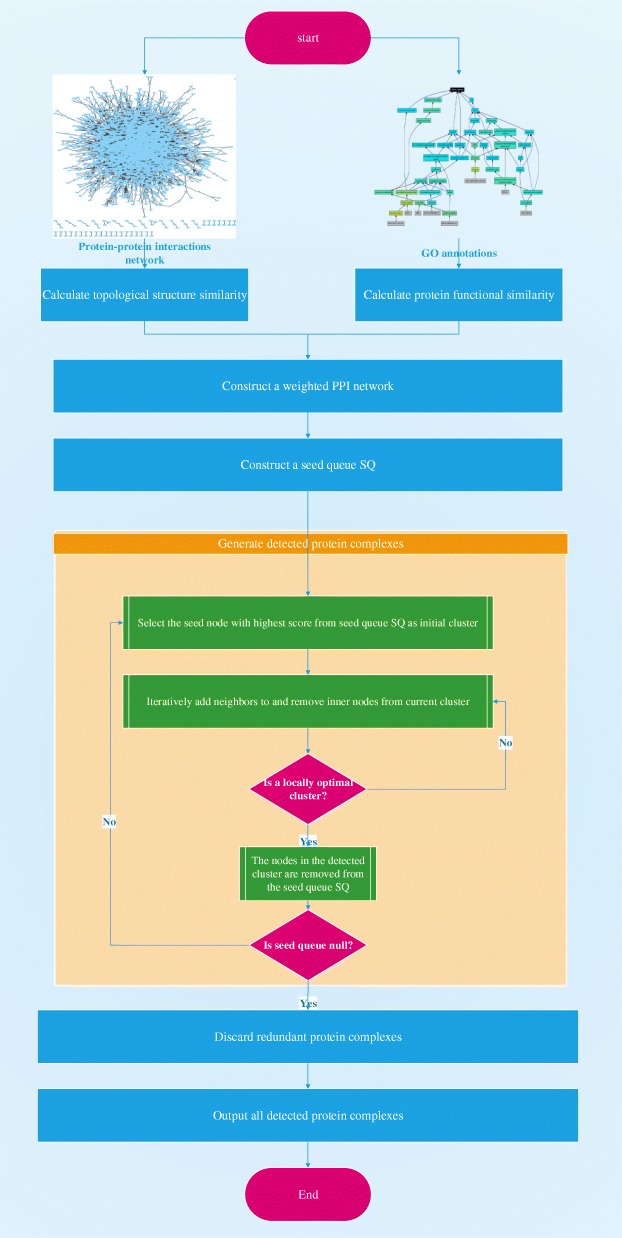
The framework of SE-DMTG

The SE-DMTG algorithm is developed to detect protein complexes based on GO annotations and PPINs topological structure. Furthermore, we propose a composite model for the identification of protein complexes. Algorithm 1 represents the main function of the proposed SE-DMTG. SE-DMTG operates in three phases. In the first step, given a PPIN, and we construct a weighted PPIN by using common neighbors and GO annotations defined by Eqs. (7) and (8). In the second step, SE-DMTG constructs a seed node queue based on a seed score function to form the initial cluster defined by Eq. (12). In the third step, based on the initial cluster in the previous step, we provide a quantitative definition of protein complexes to formulate the problem of protein complexes identification as an optimization problem defined by Eq. (17). Finally, we apply an iterative greedy search process to generate protein complexes (See Algorithm 2).False and redundancy candidate protein complexes are filtered to ultimately obtain identified protein complexes. Figure 4 shows a flowchart of SE-DMTG, which is composed of the following main steps:

1. Construct a weighted PPIN based on common neighbors and GO annotations.
2. Generate a seed queue and form an initial cluster.
3. Define the protein complex model.
4. Extend and correct the cluster to generate a locally optimal subgraph.
5. Obtain a list of identified protein complexes.

In step 1, the edge clustering coefficient probability is computed based on common neighbor via Eq. (7). The functional similarity between two proteins is calculated based on GO annotations according to Eq. (8). In step 2, we give each protein a score on the basis of both the weight degree (see Eq.(10)) and the neighborhood graph clustering coefficient (see Eq.(11)), and we sort the proteins based on their score according to Eq.(12). In step 3, we introduce a new model to estimate the quantitative value of a cluster (see Eq.(17)). In step 4, we iteratively extend and correct the cluster to generate a protein complex from the weighted PPIN. This process involves four sub-steps: selecting the highest score protein as the seed node to generate a seed queue and form the initial cluster; assessing the priority of boundary nodes in determining the priority section; iteratively adding neighbor nodes to the cluster, removing inner nodes from the cluster, and filtering and removing false candidate identified protein complex with size less than or equal to two in the extending and correcting cluster to generate a locally optimal subgraph section. In step 5, we discard some redundant candidate protein complexes and output a list of identified protein complexes. For more details of this processes, see the related sections.

### Construction of a weighted PPIN based on common neighbors and GO annotations

Recent studies [30*,*^35^*,*^36^] have shown that the accuracy of protein complex detection can be significantly improved by taking network weights into account. In the following subsections, we introduce how to calculate the weight of the PPIN.

#### Common neighbors

The edge clustering coefficient [47*] is first developed to describe how strongly neighbors are connected. However, Radicchi et al. [*47*] note that the edge clustering coefficient may not be suitable for using in PPINs because PPINs are disassortative networks. To overcome this limitation, Zhao et al. [*48*,*^49^] propose a new method to calculate the possibility of protein-protein interactions. Following their work, we also use the same method to calculate the weight of each edge, namely common neighbors (CN). Then, the existence probability of an edge (*v,u*) in a PPIN is defined as follows:

7 $$\begin{array}{@{}rcl@{}} CN(v,u)\,=\,\left\{ \begin{array}{ll} \sqrt{\frac{|N(v) \cap N(u)|^{2}}{|N(v)|\ast |N(u)|}}, & |N(v)|\!\geqslant\! 1 \ and\ |N(u)|\geqslant 1 \cr 0, &otherwise \end{array}\right. \end{array} $$

where *N*(*v*) and *N*(*u*) are the neighborhood sets of *v* and *u*, respectively. In Eq. (7), |*N*(*v*)∩*N*(*u*)| denotes the set of common neighbors between two proteins. CN is a measure that can describe how closely proteins *v* and *u* are related. In this paper, we assume that the similarity of different interactions are independent of each other. The higher the value is, the larger the probability that proteins *v* and *u* belong to the same protein complex is.

#### Protein functional similarity computation

On the other hand, from a biological perspective, gene ontology (GO) [50*] is currently one of the most comprehensive ontology databases in the bioinformatics community [*51]. The database provides a series of GO terms to describe gene product features. Proteins constituting a complex possibly have similar function. A large functional similarity means higher confidence that two proteins share similar functions. In other words, if two interacting proteins *v* and *u* have more common GO annotations and their functions are more similar, then they are more likely to belong to the same protein complex. Additionally, proteins with similar functions tend to be co-expressed [52]. Note that when two terminal nodes *v* and *u* of an edge (*v*, *u*) do not have common GO annotations, the weight of edge (*v*, *u*) may be regarded as noise and set 0.0. Here, we define a new measure to describe the similarity of two interacting proteins *v* and *u* based on a biologically similarity function defined as follows:

8 $$ {GO(\!v,u)\,=\, \left\{\begin{array}{ll} \!\frac{|GO(v) \cap GO(u)|}{\max \left(\min(|GO(v)|,|GO(u)|),Average(\!GO)\!\right)}, & \!|GO\! \cap\! GO(u)| \!>\! 0 \cr 0, &otherwise \end{array}\right.}  $$

where |*GO*(*v*)| and |*GO*(*u*)| represent the number of GO annotations in protein *v* and protein *u*, respectively. |*GO*(*v*)∩*GO*(*u*)| represents the common GO annotations for both proteins *v* and *u*. If proteins *v* and *u* share more common neighbors, the functional score is larger. Here, we use *min*(|*GO*(*v*)|,|*GO*(*u*)|) because some proteins are overlapping nodes. $Average(GO)=\frac {\sum \nolimits _{i\in V,|GO(i)|\geqslant 1}|GO(i)|}{|N|}$ is the average of the number of GO annotations for each protein in the whole PPIN. |*N*| is the number of proteins for which the number of GO annotations is greater than or equal to 1. Based on this definition, if the number of the proteins containing GO annotation is below the number of the average, then the number is adjusted to the average. max(min(|*GO*(*v*)|,|*GO*(*u*)|),*Average*(*GO*)) can penalize the reliability of edge (*v,u*) between protein *v* and protein *u* with very few GO annotations.

In this paper, SE-DMTG integrates both the topological and biological information of the PPIN by using the CN and GO. CN captures the static topological information and GO assesses the functional similarity of proteins. To incorporate both measures into our method, we use the arithmetic mean as the edge weights in the PPINs. The weight of each edge between two proteins is calculated as follows:

9 $$\begin{array}{@{}rcl@{}} w(v,u)= \left\{\begin{array}{ll} \frac{GO(v,u)+CN(v,u)}{2}, & GO(v,u)+CN(v,u) > 0 \cr 0, &otherwise \end{array}\right. \end{array} $$

Here,

1. Neighbors shared by two proteins in the network are called the common neighbors (CN) of Eq. (7).
2. The functional similarity of two proteins is quantified in terms of the GO annotation (GO) in Eq. (8).

The above two properties express the interaction based on CN and GO annotations. Note that the value of *w*(*v,u*) has a range between 0.0 and 1.0 and is used for evaluating the reliability of protein pairs to construct a weighted PPIN. The weights of each edge in the PPIN are obtained by integrating both topological information and biological information. Edges whose weights are 0.0 are considered to be noise and are deleted from the PPIN.

### Generation of a seed queue and formation of the initial cluster

Choosing high-quality protein seeds for expansion is critical. Each cluster starts at an initial cluster that consists of a single node that is generally called the seed node. An inappropriate choice of a seed node will likely affect the process of detecting protein complexes. For example, a low-quality seed node may result in a false positive protein complex being detected. Furthermore, if a protein that belongs to multiple complexes is chosen as a seed node, the resulting identified complex may subsume the multiple complexes under an unrealistically large false protein complex that cannot match any real protein complex [36*]. From a topological perspective, the central part of a protein complex often corresponds to a dense subgraph with high clustering coefficient and more reliable weight in the PPINs [*29*–*31*,*^46^*,*^53^].

According to the preliminaries section, we have given a confidence score 0≤*w*_*v,u*_≤1.0 to every edge (*v,u*)∈*E*. We utilize several measures to select seed nodes. For each node *v* in the PPIN, we define its weight degree, *d*_*w*_(*v*), as the sum of all its edge weight values:

10 $$ d_{w}(v)=\sum\limits_{(v,u)\in E}w(v,u).  $$

For each node *v*, the neighborhood graph consists of *v*, all its neighbors and the edges among them, is defined as *G*_*v*_=(*V*_*v*_,*E*_*v*_), where *V*_*v*_={*v*}∪{*u*|*u*∈*V*,(*v,u*)∈*E*} and *E*_*v*_={(*u*_*i*_,*u*_*j*_)|(*u*_*i*_,*u*_*j*_)∈*E,u*_*i*_,*u*_*j*_∈*V*_*v*_}. Futhermore, the neighborhood graph clustering coefficient (NGCC) is the sum of the weights of the edges, divided by the total number of possible edges. Thus, for a node *v*, the NGCC is defined in Eq. (11) [54]:

11 $$ NGCC(v)=\frac{\sum\nolimits_{v,u\in V_{v}}w(v,u)}{(|V_{v}|\ast(|V_{v}|-1))/2}.  $$

Here, *V*_*v*_ is the degree of node *v*, $\sum \nolimits _{v,u\in V_{v}}w(v,u)$ is the sum of the weights of the edges, and $\frac {(|V_{v}|\ast (|V_{v}|-1))}{2}$ is the total number of triangles that could pass through node *v*. The *NGCC* reflects the weight degree of aggregation of proteins in the PPINs. Note that the *NGCC* is a measure of the closeness of the node *v* and its neighbors, which varies from 0.0 to 1.0.

We devise the following score function to sort all proteins in a PPIN. If a protein has a higher score according to Eq. (12), it is more likely to be used as the seed node, to be inside a protein complex, and to have high centrality in the complexes. Thus, the score of each protein *v* is defined as the product of the its neighborhood graph clustering coefficient and its weight degree, and is defined in Eq. (12):

12 $$ Score(v)=d_{w}(v)*NGCC(v).  $$

The seed score function takes both weight degree centrality and neighborhood graph density into consideration for prioritizing the proteins for seeds. Here, we sort all proteins in the PPIN and use a queue (data structure) *SQ* to record the order. We select the highest score according to Eq. (12) as the seed node to grow a detected protein complex. Once the new detected protein complex is generated, all nodes in the detected protein complex are recorded in a list table and we choose the next highest node that is not visited in the queue *SQ* as the next seed node. Note that, we calculate the score of each protein only once based on the PPIN, which is more biological meaning [30].

### Definition of a protein complex model

As mentioned in the Background section, several protein complexes identification algorithms have been presented. Most existing algorithms make many assumptions to define a subgraph of possible protein complexes in the PPINs. However, in terms of the actual performance of these algorithms, the graphs with high density or high modularity in PPINs generally correspond to protein complexes [29*,*^35^*]. In fact, a dense graph could have low modularity, and a graph with high modularity may have low density. Therefore, the density-based algorithms ignore protein complexes with low density and the modularity-based algorithms miss protein complexes with low modularity. Overall, these methods have limitations when identifying protein complexes with various densities and modularities [*46]. To overcome these limitations, we define a new protein complex model to detect protein complexes by considering both density and modularity in the PPINs. We begin by presenting some related definitions.

According to the preliminaries section, for an undirected weighted subgraph *SG*, its density is donated as *D*_*SG*_:

13 $$ D_{{SG}}=\frac{\sum\nolimits_{(u,v)\in SG} w_{u,v}}{|SG|*(|SG|-1) /2}  $$

where $\sum \nolimits _{u,v\in SG} w_{u,v}$ is the sum weight of the edges contained in subgraph *SG*, and |*SG*| represents the size of the subgraph *SG*, respectively. The density of a graph measures how close the graph is to a clique, and the density takes value between 0.0 and 1.0.

For the subgraph *SG*⊆*G*, its weighted in-degree, denoted as $d_{w}^{in}(SG)$, is the sum of the weights of all edges belonging to *SG*, and its weighted out-degree, denoted as $d_{w}^{out}(SG)$, is the sum of the weights of the edges connecting the nodes in *SG* to the nodes in the rest of graph *G*. $d_{w}^{in}(SG)$ and $d_{w}^{out}(SG)$ can be obtained as follows [46]:

14 $$ d_{w}^{in}(SG)=\sum\limits_{u,v\in SG;(u,v)\in E}w(u,v).  $$

15 $$ d_{w}^{out}(SG)=\sum\limits_{v\in SG;u\notin SG;(u,v)\in E}w(u,v).  $$

Clearly, the weighted degree of *d*_*w*_(*SG*) is equal to the sum of $d_{w}^{in}(SG)$ and $d_{w}^{out}(SG)$.

The modularity *M*_*SG*_ of a subgraph *SG*⊆*G* is defined as follows:

16 $$ M_{{SG}}=\frac{d_{w}^{in}(SG)}{d_{w}^{in}(SG)+d_{w}^{out}(SG)}.  $$

Obviously, *M*_*SG*_ takes values from 0.0 to 1.0. If a subgraph has higher modularity, it has more connections within itself and fewer connections to the rest of the PPIN. A subgraph with a modularity of 1.0 has no connections with the rest of the PPIN.

In this model, in the process of identifying protein complexes, we measure the quality of *SG* by considering its density (*D*_*SG*_) and modularity (*M*_*SG*_). *D*_*SG*_ describes the density of subgraph *SG*, *M*_*SG*_ describes the modularity of subgraph *SG* and $\sqrt {D_{{SG}}*M_{{SG}}}$ describes the subgraph with both high density and high modularity. Here, to make the value range of a subgraph with both high density and high modularity the same as that of the density and modularity, i.e, [0.0,1.0], the value of *D*_*SG*_∗*M*_*SG*_ is normalized by the geometric mean of *D*_*SG*_ and *M*_*SG*_. The fitness of a subgraph *SG* in an undirected weighted graph *G*, denoted as *F*(*SG*), is defined as:

17 $$ F(SG)=\frac{D_{{SG}}+M_{{SG}}+\sqrt{D_{{SG}}*M_{{SG}}}}{3}.  $$

Generally, as the subgraph *SG* expands, its modularity increases and its density decreases. Thus, by expanding from a node, we can obtain a subgraph with the local maximum fitness score and output the result as a protein complex. Thus, this new model can be used for identifying protein complexes with different topology, including high density but low modularity, high modularity but low density, and high density and high modularity. Therefore, our model can identify the protein complexes with various densities and modularities.

### Extending and correcting the cluster to generate a locally optimal subgraph

#### Determining the priority of boundary nodes

An initial cluster (*SG*) starts as single protein, and then grows and shrinks gradually as proteins are added and removed one by one. The process of adding proteins from the neighbor of *SG*, and is denoted as *Neighbor*(*SG*), and the process of removing proteins from the inner nodes is denoted as *inner*_*nodes*(*SG*). In this process, we first define two concepts: if *p*∈*Neighbor*(*SG*), the neighbor node connects to at least one edge with any protein of cluster *SG* but does not belong to *SG*; If *p*∈*inner*_*nodes*(*SG*), the inner node belongs to *SG*, but connects to at least one node which is a neighbor of *SG*. A key problem is to decide the priority to add and remove proteins in terms of *SG*. In general, if a protein *v* belongs to *SG*, it may have a strong connection with its cluster *SG*=(*V*_*SG*_,*E*_*SG*_). Therefore, if the protein *v* is added to *SG*, it could increase the average of the weighted interactions within *SG*. By contrast, if the protein *v* is removed from *SG*, it could increase the average of the weighted interactions within *SG*. Here, we introduce a measure to assess the priority, denoted as *weight*_*avg*_(*SG*), which is defined as:

18 $$ weight_{{avg}}(SG)=\frac{2*\sum\nolimits_{(v,u)\in E_{{SG}}} weight(v,u)}{|V_{{SG}}|},  $$

where *weight*_*avg*_(*SG*) is the average of the weighted interactions of all proteins within *SG*, |*V*_*SG*_| is the number of proteins in *SG* and $\sum \nolimits _{(v,u)\in E_{{SG}}} weight(v,u)$ represents the total weight of the interactions in *SG*. The priority of adding the node *p* into the cluster SG, where *p*∈*Neighbor*(*SG*), or deleting the node *p* from the cluster SG, where *p*∈*inner*_*nodes*(*SG*), *SG* is determined by the value of *weight*_*avg*_(*SG*). We choose the highest *weight*_*avg*_(*SG*) of the boundary node to add it to *SG* or remove it from *SG* to maximize the value of *F*(*SG*) (see Eq.(17)).

#### Extending and correcting estimation

For a cluster *SG*, in extending step, we first obtain all the neighbors, namely, *Neighbors*(*SG*). The priority of all neighbors is determined by the value of *weight*_*avg*_(*SG*) see Eq. (18). Whether the highest priority protein *v* is added to *SG* is determined by whether the fitness (*F*(*SG*)) of *SG* is increased after the highest priority protein *v* is added and whether the actual edge between the highest priority protein *v* and the *SG*, denoted as |*SG*∩*N*(*v*)|, which is the number of proteins in *SG* connected with *v* is greater than the expectation edge, denoted as *F*(*SG*)∗|*SG*|, where *F*(*SG*) is the fitness of *SG* and |*SG*| is the number of proteins in *SG*. Once the highest priority protein *v* is added to *SG*, *SG* is updated, i.e., the highest priority protein *v* is removed from *Neighbors*(*SG*). Then, the next highest priority protein is tested, and the priorities of list *Neighbors*(*SG*) and the fitness (*F*(*SG*)) of *SG* are recalculated, and so on. If the highest priority protein *v* fails any of two tests, then *SG* cannot be further extended.

For a cluster *SG*, in the correcting step, we first obtain all inner nodes, namely *Inner*_*nodes*(*SG*). The priority of all proteins in *Inner*_*nodes*(*SG*) is determined by the value of *weight*_*avg*_(*SG*) (see Eq. (18)). Whether the highest priority protein *v* is deleted from *SG* is determined by whether the fitness (*F*(*SG*)) of the cluster *SG*−{*v*} is increased after the highest priority protein *v* is removed from *SG* and whether the actually edge between the highest priority protein *v* and *SG*−{*v*}, denoted as |*SG*−{*v*}∩*N*(*v*)|, which represents the number of proteins in *SG*−{*v*} connected with *v*, is greater than the expectation edge, denoted as *F*(*SG*)∗|*SG*|, where *F*(*SG*) is the fitness (*F*(*SG*)) of *SG* and |*SG*| is the number of proteins in *SG*. Once the highest priority protein *v* is removed from *SG*, the cluster *SG* is updated, i.e., the highest priority protein *v* is removed from *Inner*_*nodes*(*SG*). Then, the next highest priority protein is tested, and the priorities of *Inner*_*nodes*(*SG*) and the fitness of the cluster *SG*−{*v*} are recalculated, and so on. If the highest priority protein *v* fails any of two tests, then the cluster *SG* cannot be further corrected.

### Obtaining a list of identified protein complexes.

On the basis of the quantitative description of protein complexes, we develop a novel clustering algorithm based on density and modularity with network topology and GO annotations, named SE-DMTG, to identify protein complexes in a weighted PPIN whose edge weights reflect the reliability of the edge in a protein complex according to topological and biological information.

The input of the SE-DMTG algorithm is a PPIN, which is described as a simple undirected graph *G*(*V,E*) with GO annotations. The SE-DMTG algorithm broadly consists of four phases. First, SE-DMTG constructs a weighted PPIN-based topological and biological information at lines 2-11. Second, SE-DMTG calculates the scores of all nodes and selects the node with the maximum score as the seed in lines 12-18. Third, starting from the seed node, a greedy procedure is used for adding nodes to or removing nodes from the cluster *SG* to obtain a subgraph with high graph fitness. The growth process is repeated from different seeds to form multiple, possibly overlapping subgraphs in lines 19-49. Once a new cluster is completed, all nodes in this cluster *SG* are recorded to prevent them from being used as seed nodes. Then, we select the next seed node from those remaining in the queue *SQ* to generate the next cluster *SG* in lines 41-45. Moreover, we discard candidate complexes whose size is less than 3 [35] and remove unreliable candidate complexes at line 38-46. Finally, we discard redundant protein complexes in lines 50-55. A detailed description of the SE-DMTG algorithm is shown in Algorithm 1.


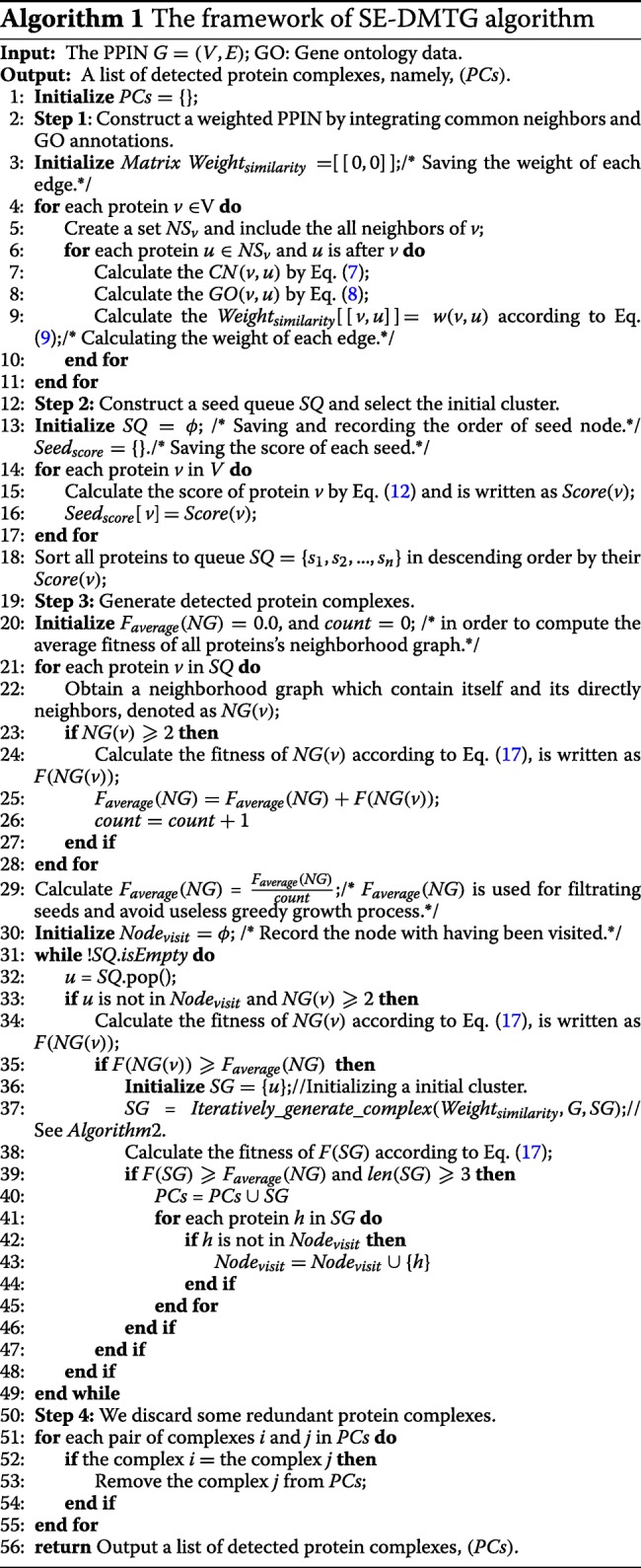


In the first step, we assign a weight to each edge based on common neighbor and gene ontology data (lines 2 ∼11).

In the second step, SE-DMTG calculates the score of each node (lines 12 ∼17). Furthermore, all the nodes in network *G* are queued into *SQ* in non-increasing order of *Score*(*v*) (line 18).

In the third step, we choose the node with the highest *Score*(*v*) that has not yet been visited before to bring it up (lines 19 ∼29). The key idea of this step is that any neighbors of the current subgraph *SG* that make a positive contribution to *F*(*SG*) will be added to *SG* or removed from *SG* (line 37). The description of iterative generation of a complex is shown in Algorithm 2. Algorithm 2 has two subphases, and we can gradually add neighbors to cluster *SG* or remove inner nodes from cluster *SG*. As for the priority of candidate nodes is based on (see Eq. (18)) and two conditions. More details are introduced in the section on extending and correcting the cluster to generate a locally optimal subgraph.

Next the step-by-step procedure of step 3 is given in Algorithm 2.


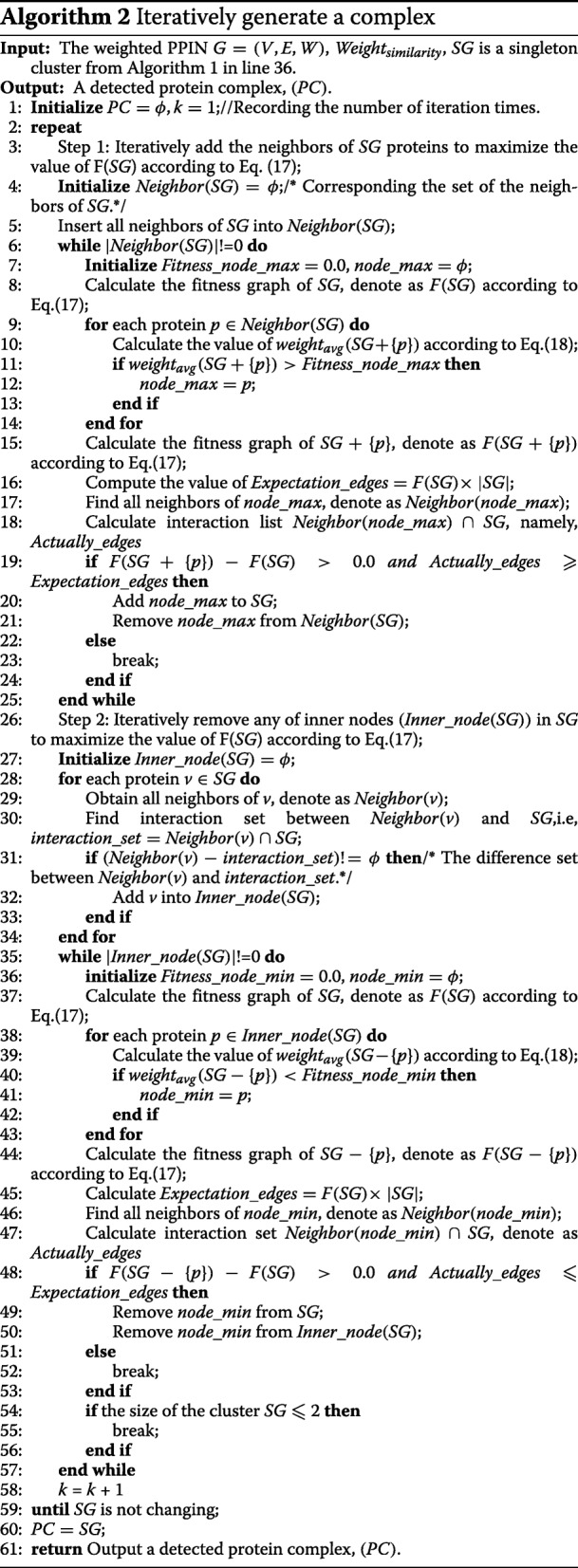


In the first phase in lines 3 ∼25, after obtaining a seed protein, we first get an external boundary protein set that consists of the neighbors of *SG* called *Neighbor*(*SG*), in lines 4 ∼5. Then, we calculate the graph fitness of *SG* at line 8. Furthermore, we find the neighbor protein with the highest priority according to *weight*_*avg*_(*SG*+{*p*}) in *Neighbor*(*SG*), which is added to *SG* to maximize the value of *weight*_*avg*_(*SG*+{*p*}) in lines 7 ∼14. Furthermore, we calculate the fitness of graph *SG*+{*p*} in line 15, and *Expectation*_*edges* is calculated according to the graph fitness of *SG* × the size of *SG* in line 16. Meanwhile, we also calculate the value of *Actually*_*edges* which is the size of the interaction set between *Neighbor*(*node*_*max*) and *SG*, denoted as *Neighbor*(*node*_*max*)∩*SG*, in line 18. If the *node*_*max* with the highest priority is added to increase the value of *F*(*SG*) and the *Actually*_*edges* is larger than *Expectation*_*edges*, then we add *node*_*max* to *SG* and remove it from *Neighbor*(*SG*) in lines 19 ∼24. We continually check the next highest priority node in *Neighbor*(*SG*) and judge whether the node can be added to the *SG* in lines 6-25. Otherwise, the iterative addition of the neighbors of *SG* phase is terminated when one of two conditions is not satisfied in line 19 or when no more remaining neighbor nodes can be added to *SG* in line 6.

In the second phase, SE-DMTG allows the removal of any inner nodes in cluster *SG* to maximize the value of *F*(*SG*) in lines 26 ∼57. We first find the inner nodes that have edges with nodes that are not in *SG*, denote as *Inner*_*node*(*SG*) in lines 27 ∼34, and then we test whether each node in *Inner*_*node*(*SG*) can be removed from *SG* in lines 35-57. We first find the highest priority node according to Eq. (18) in lines 36-43. Meanwhile, we calculate the graph fitness *F*(*SG*−{*p*}) of *SG*−{*p*} in line 44. Similarly, we calculate the values of *Expectation*_*edges* and *Actually*_*edges* in lines 45 ∼47. If the two conditions in line 48 are satisfied, we remove the node from *SG* and *Inner*_*node*(*SG*) in lines 49 ∼50; otherwise, the second phase is terminated in lines 51 ∼57.

In Algorithm 2, the key idea is to iteratively add the highest priority node in *Neighbor*(*SG*) to the cluster *SG* or remove the highest priority node in *Inner*_*node*(*SG*) from the cluster *SG* to maximize the value of graph fitness *F*(*SG*) in lines 2 ∼59. This growth process is repeated until the current cluster *SG* no longer changes and is a locally optimal subgraph in line 59; then, the detected protein complex is output by Algorithm 1 in line 37.

After we obtain a detected complex *SG* by using Algorithm 2 in line 37, and we discard fake protein complexes and complexes whose size is less than 3 [35] in line 39. As a result, we save the detected complex *SG* in line 40. Meanwhile, SE-DMTG records the nodes in *SG* in lines 41 ∼45 and selects the next seed node by considering the rest of nodes in seed queue *SQ* that have not been included in any of the detected complexes found thus far. The next node with the highest score is selected as the seed (lines 31 ∼35). We recursively perform the above key operations in PPIN to identify the remaining candidate protein complexes until no seed nodes remain in seed queue *SQ* (lines 31-49). Note that when this process is repeated, the nodes in the previously generated protein complex remain in the PPIN; therefore, SE-DMTG is able to generate overlapping complexes.

Finally, SE-DMTG outputs all identified protein complexes in line 56.

## Data Availability

The datasets used and/or analysed during the current study are available from the corresponding literatures and datasets.

## Abbreviations

BP: Biological process
CC: Cellular component
ClusterONE: Clustering with overlapping neighborhood expansion
CMC: Clustering-based on maximal cliques
CN: Common neighbors
Co-IP: Co-immunoprecipitation
GO: Gene ontology
GO: GO annotations (gene ontology)
MCL: Markov clustering
MCODE: Molecular complex identification
MF: Molecular function
PPINs: Protein-protein interaction networks
SE-DMTG: A seed-extended algorithm for detecting protein complexes based on density and modularity with topological structure and GO annotations
SQ: Seed queue
TAP-ms: Tandem affinity purification with mass spectrometry
